# Elucidating the primary mechanisms of high-intensity interval training for improved cardiac fitness in obesity

**DOI:** 10.3389/fphys.2023.1170324

**Published:** 2023-08-07

**Authors:** Bing Bo, Aijing Guo, Severa Jafeth Kaila, Zhe Hao, Huiqing Zhang, Jianshe Wei, Yuan Yao

**Affiliations:** ^1^ Department of Kinesiology, School of Physical Education, Henan University, Kaifeng, China; ^2^ Sports Reform and Development Research Center, School of Physical Education, Henan University, Kaifeng, China; ^3^ Institute for Brain Sciences Research, School of Life Sciences, Henan University, Kaifeng, China

**Keywords:** high-intensity interval training, obesity, cardiac fitness, adipose tissue, inflammation

## Abstract

Obesity is a global and rising multifactorial pandemic associated with the emergence of several comorbidities that are risk factors for malignant cardiac remodeling and disease. High-intensity interval training (HIIT) has gained considerable attention due to its favorable outcomes of cardiometabolic health in individuals with overweight or obese. The primary aim of this review is to discuss the fundamental processes through which HIIT improves cardiac impairment in individuals with obesity to develop viable treatments for obesity management. In this review, a multiple database search and collection were conducted from the earliest record to January 2013 for studies included the qualitative component of HIIT intervention in humans and animals with overweight/obesity related to cardiac remodeling and fitness. We attempt to integrate the main mechanisms of HIIT in cardiac remolding improvement in obesity into an overall sequential hypothesis. This work focus on the ameliorative effects of HIIT on obesity-induced cardiac remodeling with respect to potential and pleiotropic mechanisms, including adipose distribution, energy metabolism, inflammatory response, insulin resistance, and related risk profiles in obesity. In conclusion, HIIT has been shown to reduce obesity-induced risks of cardiac remodeling, but the long-term effects of HIIT on obesity-induced cardiac injury and disease are presently unknown. Collective understanding highlights numerous specific research that are needed before the safety and effectiveness of HIIT can be confirmed and widely adopted in patient with obesity.

## 1 Introduction

Obesity, characterized by the condition of being grossly fat or overweight, is a pandemic that has received extensive medical attention (Piche et al., 2020). Approximately 2.8–3.5 billion people, 39%–49% of the world’s population, are affected by overweight or obesity (Maffetone et al., 2016). According to the global burden of disease research encompassing 195 nations, over 603.7 million adults and 107.7 million children suffer from obesity (Collaborators et al., 2017). Around four million deaths induced by obesity and greater than two-thirds of this number is attributed to cardiovascular disease (Collaborators et al., 2017). Obesity has been formally designated as a significant and independent risk factor for cardiac remodeling by the American Heart Association (Arnett et al., 2019; Hingorani et al., 2020; Powell-Wiley et al., 2021).

Adipose tissue (AT) integrity and functioning are essential factors determining cardiometabolic risk throughout obesity development. Hypoxia, fibrosis, inflammation, dysregulated adipokine secretion, and impaired mitochondrial function are all local effects of AT expansion. The systemic effects include insulin resistance (IR), aberrant glucose and lipid metabolism, hypertension, and a pro-inflammatory state, all of which provide linking mechanisms between obesity and cardiac structural and functional impairment (Ren et al., 2021).

A growing body of evidence has demonstrated that exercise exerts beneficial effects on weight loss in obesity (Pedersen et al., 2019; Bellicha et al., 2021). High-intensity interval training (HIIT) is an effective intervention to ameliorate metabolic disturbances, inflammation, IR, and related risk profiles in the heart during obesity development (Bluher et al., 2017; Ratajczak et al., 2020; Vaccari et al., 2020; Paahoo et al., 2021). This review summarizes the primary mechanism by which HIIT improves cardiac function in obesity. We also highlight HIIT as an efficient potential non-pharmacological therapeutic strategy for managing obesity-induced cardiac damage. For this study, a multiple database search and collection were conducted from the earliest record to January 2013 for studies included the qualitative component of HIIT intervention in humans and animals with overweight/obesity related to cardiac remodeling and fitness.

## 2 Cardiac structural and functional damage in obesity

**FIGURE 1:**
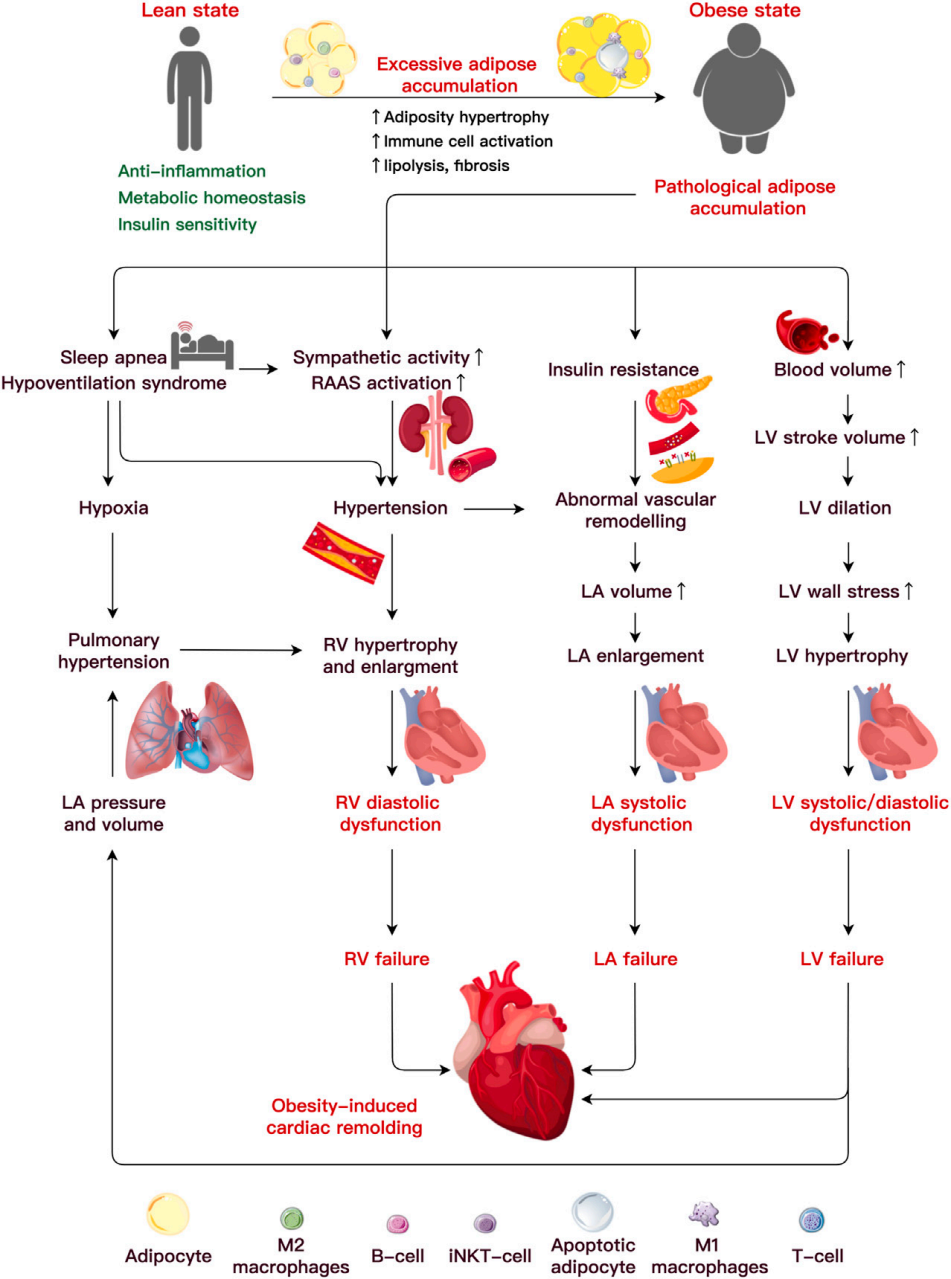
Etiology underlining obesity-induced cardiac remodeling. Under pathological adipocyte accumulation, AT dysfunction and inflammation bring about abnormalities in the cardiac hemodynamics, which lead to alterations in ventricular function and heart failure. An increased blood volume induces left ventricular (LV) hypertrophy and systolic and diastolic dysfunction. It is uncertain how metabolic or functional alterations, including insulin resistance, elevated sympathetic activity, altered renin-angiotensin-aldosterone system (RAAS), and sleep apnea, may lead to LV or RA failure. Abbreviations: iNKT cells, invariant natural killer T cells; LA, left atria; LV, left ventricular; RV, right ventricular.

Obesity causes hemodynamic changes that predispose to ventricular structural and functional damage, including increased blood pressure, stroke volume (SV), cardiac output (CO), and increased left ventricular (LV) and left atrial (LA) filling pressures. These factors primarily result in unfavorable ventricular structure and function, contributing to the etiology of obesity-related cardiac remodeling (Feng et al., 2019; Robertson et al., 2020; Lewis et al., 2021) (Figure 1).

Individuals with obesity are often at a high risk of LV hypertrophy, defined by an increase in the ventricular mass (Fumagalli et al., 2020). The prevalence of LV hypertrophy is 12% in individuals with obesity and 78% in subjects affected by severe obesity (Avelar et al., 2007), and both are much higher than in their lean counterparts (2%) (Finocchiaro et al., 2018). In contrast to obesity [body mass index (BMI) > 30 kg/m^2^], severe obesity (BMI>35 or 40 kg/m^2^) carries a greater risk of serious health complications. Data collected from 826 participants showed that central obesity (reference waist-to-hip ratio and waist-to-height ratio) is the primary independent risk factor that accounted for 66.3% of subjects with LV mass increase and 31.2% of participants with LV geometry worsening even after adjusting BMI (Lee et al., 2019). A recent study argued that abdominal adipocyte accumulation increased the arterial load, likely due to concentric LV remodeling (Mandry et al., 2021). Correspondingly, the LV structural alteration raises the risk of systolic and diastolic dysfunction in the heart (Neeland et al., 2018). After a multivariate correction, a significant connection between the BMI and the incidence of clinical hypertrophic cardiomyopathy (HCM) (with a hazard ratio of 1.063 for every 1 kg/m^2^ rise in BMI) indicated that obesity seems to modify the phenotypical expression of HCM (Park et al., 2020). Elevated BMI and IR increased the risk of future heart failure (HF) with preserved ejection fraction (HFpEF) by more than three times compared to a reduced ejection fraction (Savji et al., 2018). Moreover, increased visceral (Neeland et al., 2013; Rao et al., 2020) and epicardial fat (Elsanhoury et al., 2021) had a significantly stronger association with the HFpEF risk than increased subcutaneous fat.

AT accumulated around the atria is often correlated with atrial structural and functional alterations. One 16-year longitudinal tracking study of 4,403 Framingham participants (mean age 45 years, 48% male) revealed that BMI is the pivotal risk factor associated with LA enlargement (McManus et al., 2010). The LA anterior-posterior diameter and LA volume increase in severe obesity (mean age 47 years, 21% male) (Litwin et al., 2020). Additionally, LA enlargement occurs concurrently with LV expansion and mirrors eccentric LV remodeling in patients with obesity (mean age 50 years, 48% male) (Aiad et al., 2019). The increased LA diameter and volume induced by obesity can induce atrial fibrillation (AF) (Kanazawa et al., 2014; Feng et al., 2019). A meta-analysis of 51 trials including 626,603 individuals found that each five-unit increase in the BMI increased the probability of the incident AF by 19%–29%, post-operative AF by 10%, and post-ablation AF by 13% (Wong et al., 2015). As a result, obesity is linked to LA remodeling, a process in which the structure, electrophysiological function, and electro-anatomical integrity of the atrium gradually deteriorate, raising the risk of pro-arrhythmia and cardiac disease (von Haehling et al., 2019; Walczak-Galezewska et al., 2019; Chien et al., 2021).

Obesity is a risk factor for right ventricular (RV) hypertrophy and dysfunction on its own (Ren et al., 2021). Indeed, the RV mass was increased by 6% and 14% in the groups with overweight and obesity (mean age 61.5 years, 47% male), respectively. The researchers noticed that for every 5 kg/m^2^ increase in the BMI, the RV mass increased by 1.3 g, and the RV end-diastolic volume (RVEDV) increased by 8.65 mL (Chahal et al., 2012). The RVEDV, RV ejection fraction, and RV diastolic filling are impaired by obesity (Lewis et al., 2021). A recent study revealed a substantial link between visceral fat accumulation and decreased RV longitudinal strain, which may contribute to obesity increased risk of HF (Sawada et al., 2020). Another strain investigation using feature tracking verified that severe obesity impairs the LA reservoir and atrial contraction phases, implying an early loss of the atrial contraction compensatory capacity in the severely obese (aged 7–18 years, 45% male) (Xu et al., 2021). Both obstructive sleep apnea (OSA) and pulmonary arterial hypertension are associated with the variables contributing to RV remodeling and damage in obesity (Adir et al., 2021). The hallmark of OSA is recurring complete and partial upper airway obstructions, which cause intermittent hypoxia and fragmented sleep (Yeghiazarians et al., 2021). BMI, waist circumference and neck size are all associated with an increased risk of OSA (Peppard et al., 2000). Obesity is linked to OSA include fat deposits in the upper airway and a decrease in lung volume leads to less caudal traction on the upper airway (Jordan et al., 2014). The molecular characteristics of OSA are oxidative stress, upregulation of redox-sensitive genes, inflammatory cascade, and increased catecholamines, which are all crucial cardiovascular disease mediators. OSA prevalence ranges from 40% to 80% in patients with hypertension, AF, HF, coronary artery disease, pulmonary hypertension, and stroke (Javaheri et al., 2017).

Additionally, intermuscular adipose tissue (IMAT) refers to the fatty deposits found between and within muscles. It can accumulate due to age, obesity, and metabolic diseases like diabetes. IMAT secretes inflammatory cytokines, which can cause blood vessel damage and contribute to atherosclerosis (Addison et al., 2014; Goodpaster et al., 2023). Increased IMAT is linked to ectopic adipose deposition in organs like the heart, which account for numbers of cardiovascular risk factors, including insulin resistance, inflammation, atherosclerosis, and arterial stiffness (Yim et al., 2007). Higher IMAT levels predict incidence of cardiovascular events and mortality independent of general obesity by impair the cardiac structure and function (Yim et al., 2007).

Furthermore, the remodeling of the heart associated with obesity is characterized by the progressive replacement of the myocardium by irregular accumulation of AT that can separate and result in pressure-induced atrophy of the myocardial cells (Poirier et al., 2006). Moreover, obesity-induced degenerative remodeling and impairment of the heart appears to be progressive and irreversible, implying a poor clinical prognosis.

## 3 What is HIIT?

A constellation of studies revealed that weight loss is critical for preventing and managing the cardiac damage through lifestyle modifications (Liu et al., 2022) and regular physical activity in obesity (Sultana et al., 2019; Bellicha et al., 2021). HIIT is a dynamic and complex training plan that has grown in popularity and become a fitness trend over the past three decades (Tremblay et al., 1994; Sultana et al., 2019; Bull et al., 2020). HIIT, in contrast to moderate-intensity continuous training (MICT), is defined by repeated short bouts of high-intensity exertion [≥90% of the maximal oxygen consumption (VO_2_max) for healthy subjects or ≥80% VO_2_max for clinical populations] interspersed by low-intensity or passive recovery (Buchheit and Laursen, 2013). Also distinct from sprint interval training (SIT), which is characterized by “all-out” or “supramaximal” efforts (>100% VO_2_max) in studies with healthy subjects, HIIT is described as “near maximal” efforts typically performed at an intensity that elicits ≥80% VO_2_max (but frequently 85%–95%) of maximal heart rate (Weston et al., 2014; Atakan et al., 2022). Thus, this present narrative review focuses primarily on data obtained from HIIT intervention in obesity-induced cardiac remolding based on exercise intensity. Additionally, the prescription of HIIT is also dynamic and sophisticated because it permits the guidance of a number of variables, such as exercise type, intensity, duration, and volume. HIIT improves VO_2_max, cardiometabolic health, endothelial function, resting blood pressure, metabolic capacity, and upregulates antioxidant capacity. Compared to MICT, HIIT provided a 28.5% larger decrease in the total fat mass and exerted more beneficial effects on cardiac fitness improvement in subjects with overweight or obesity (Viana et al., 2019; Vaccari et al., 2020). However, excessive training volume of HIIT might impair mitochondrial function in skeletal muscle (Flockhart et al., 2021) and increase the risks of metabolic side-effects (Alkhatib et al., 2020). Admittedly, the sufficient evidence of dose-response correlations required to achieve the optimal advantages of HIIT is still lacking at this time (Joisten et al., 2022). Despite the controversial effect of HIIT, several studies of humans (Racil et al., 2016a; Racil et al., 2016b; Tong et al., 2018; Christensen et al., 2019; Sun et al., 2019; Vaccari et al., 2020; Reljic et al., 2021a; Hearon et al., 2022; Poon et al., 2022; Reljic et al., 2022; Youssef et al., 2022) and rodents (de Oliveira Sa et al., 2017; Kim et al., 2018; Boardman et al., 2019; Franca et al., 2020; Astani et al., 2022) demonstrated that HIIT is an efficient strategy for health and fitness improvement in obesity due to its remarkable benefits in adipocyte distribution, AT microenvironment, inflammation, IR, and other obesity-induced cardiac deteriorations.

## 4 HIIT ameliorates cardiac structure and function in obesity

Numerous studies have suggested that HIIT exerts predominant effects on improving cardiac structure and function in the obese heart. After 3 months of high-intensity running (90% VO_2_max, 3 sessions/week), the LV wall thickness presented with a reduction of approximately 6.5% in high-fat feeding male mice (12 weeks old) (de Oliveira Sa et al., 2017). In the ischemic myocardium exposed to a high-fat load, HIIT also displayed practical cardioprotective effects through cardiac function amelioration. For instance, high-intensity treadmill running (85%–90% VO_2_max, 25^o^ inclination, 5 sessions/week) considerably elevated the recovery of the maximum and minimum pressure derivative (dP/dtmax and dP/dtmin) without heart rate (HR) alteration, as well as LV development pressure in ischemic insult mice with obesity (Lund et al., 2015; Boardman et al., 2017; Boardman et al., 2019). These findings revealed that HIIT reduced post-ischemic stunning and increased LV contraction by ameliorating contractility and relaxation in obesity. VO_2_max is the most important representative indicator of cardiopulmonary function, and numerous studies have indicated that HIIT effectively increased VO_2_max in humans (walking, 100% VO_2_max, 3 sessions/week) (Vaccari et al., 2020) and rodents (85%–90% VO_2_max, 25^o^ inclination, 5 sessions/week) (Boardman et al., 2017) affected by overweight or obesity. Although the training regimes are variable and complex, HIIT excels at promoting aerobic capacity in obesity. It has been demonstrated that 5 weeks of HIIT cycling (85% ± 7% VO_2_max, 4 sessions/week) positively affects VO_2_max improvement in Chinese young women (aged 18–30 years) with obesity (Kong et al., 2016a). Eight weeks of high-intensity interval cycling (100% maximum power output, 3 sessions/week) could significantly increase the VO_2_max in adults (mean age 54 years, 81% male) with obesity (Mendelson et al., 2022). If the training period is extended to 12 weeks, HIIT (100% maximum power output, 3 sessions/week) is more effective than MICT at boosting VO_2_max than MICT in adolescent affected by obesity (Cao et al., 2022; Diniz et al., 2022). In the elderly (mean age 67.7 years) with obesity, 3 months of high-intensity interval elliptical cross-training (80%–85% HRmax, 3 sessions/week) also could increase 6-min walking test (6MWT) (Youssef et al., 2022). Among the patients with obesity in stage A HF, 12 months of HIIT (95% HRmax, 3 sessions/week) increased LV end-diastolic volume (LVEDV) and VO_2_max to improve the pathophysiologic characters of HFpEF (Hearon et al., 2022). Even 4 days of HIIT training (80% VO_2_max, 4 sessions/week) was sufficient to maintain myocardial contractility and relaxation following a cardiac insult in high-fat-fed male rats (6 weeks old) (Franca et al., 2020). Monitoring cardiac structure and functional changes resulting from obesity development could provide significant insights into revealing a more intuitional picture of the exercise-induced effects. However, it is important to note that the duration of HIIT in the above studies did not exceed 12 weeks, which does not provide substantial evidence for the long-term effects of HIIT on obesity-induced structural and functional remodeling of the heart. In the following section, we will summarize the primary mechanisms of HIIT in obesity-induced cardiac remodeling management to reveal the clue that HIIT may be a better tool for cardiac disease improvements programs that need to be sustainable in obesity.

## 5 Primary mechanisms of HIIT in cardiac fitness improvements in obesity

**FIGURE 2:**
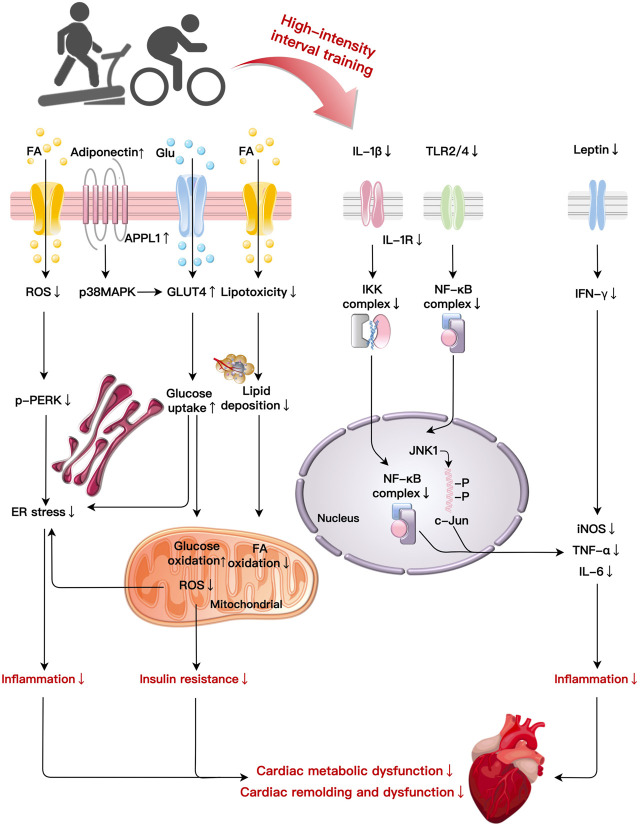
Primary mechanisms of HIIT in cardiac fitness improvement in obesity: HIIT ameliorates cardiac metabolic dysfunction and remodelling induced by obesity through multiple basic mechanisms such as improvements in lipotoxicity, glucose metabolism, inflammation, and ER stress. HIIT reduce lipid deposition, FA oxidation, and ROS production. HIIT decreases p-PERK to reduce ER stress-related inflammation while elevates glucose uptake and oxidation to improve insulin resistance induced by obesity. HIIT suppresses the production of pro-inflammatory cytokines (IL-1β) via NF-κB signaling. Decreases in IL-1β induced by HIIT also mitigate IKK and NF-κB complexes leading to inflammation. After HIIT intervention, TLR2/4 reduce inflammation by NF-κB complexes and the JNK1/c-Jun pathway, respectively. HIIT decreases leptin and IFN-γ expression to alleviate the inflammatory reaction. Abbreviations: c-Jun, a transcription factor; ER, endoplasmic reticulum; FA, fatty acid; Glu, glucose; GLUT4, glucose transporter type 4; IKK, I-κB-kinase; JNK1, C-Jun NH2-terminal kinase 1; NF-κB, nuclear factor κB; PERK, protein kinase RNA-like ER kinase; TLR, toll-like receptor.

Chronically exceeding necessary calorie intake promotes obesity and the development of cardiac remodeling and damage. As a therapeutic intervention, HIIT could improve AT distribution, metabolic abnormalities, inflammatory response, and IR to ameliorate obesity-induced cardiac impairment. In this section, we attempt to integrate the main mechanisms of HIIT in cardiac remolding improvement in obesity into an overall sequential hypothesis (Figure 2; Table 1).

**TABLE 1:** The effects of HIIT on obesity-induced cardiac damage improvements in rodent and human studies.

Classification	Factor	Species	*N*	Subject information	Exercise model	Duration	Observation	References
AT distribution	VAT	Human	46	WWO BFP >30 (age, 18–23 years)	Cycling: HIIT regime consisted of 90% VO_2max_ for 4 min, passive recovery (pedal frequency, 60 rpm) for 3 min, target work 400J/per session	12 weeks 3–4 sessions/week	VAT↓, VO_2max_↑	Tong et al. (2018)
	VAT	Human	43	WWO BMI>25 kg/m^2^ BFP >30 (age, 18–22 years)	Cycling: HIIT regime consisted of 90% VO_2max_ for 4 min, passive recovery (pedal frequency, 60rpm) for 3 min, target work 300J/per session	12 weeks 3 sessions/week	VAT↓, VO_2max_↑	Zhang et al. (2017)
	EAT	Human	39	Inactive adults (26% males) with abdominal obesity (age, 41 ± 14 years)	Cycling: HIIT regime consisted of 6 bouts 75%–85% VO_2max_ for 2 min, passive recovery at 30% VO_2max_ for 1 min	12 weeks 3 sessions/week	EAT↓, VO_2max_↑	Christensen et al. (2019)
	EAT	Human	34	PWO (52.9% male) with hypertensive BMI>24 kg/m^2^ (age, 50.9 ± 7.9 years)	Treadmill running: HIIT training consisted in 5 bouts at 80% HRR for 3 min, active recovery at 40% HRR for 3 min	8 weeks 3 sessions/week	EAT↓, SBP↓, DBP↓, TG↓	Jo et al. (2020)
	EAT	Human	28	Healthy subjects and subjects with DGT BMI = 27.9–31.7 kg/m^2^ (age, 43–53 years)	Cycling: HIIT regime consisted of 4–6 bouts at maximal all-out for 30s, 4 min recovery between each bout (load, 10% of LBM)	2 weeks 3 sessions/week	EAT↓, PAT↓, VO_2max_↑, insulin sensitivity↑	Honkala et al. (2017)
Microenvironment	Mitochondrial respiration	Human	32	PWO mean BMI = 36 kg/m^2^ (mean age, 39 years)	Treadmill walking: HIIT training consisted in 3–7 bouts at 100% VO_2max_ for 3 min, interval walking at 50%VO_2max_ for 1.5 min	12 weeks 3 sessions/week	Fat oxidation rate↑, ADP-stimulated mitochondrial respiration↑, VO_2max_↑	Vaccari et al. (2020)
	Oxygen sparing	Mice (C57BL/6)	42	Male (age, 5–6 weeks) High-fat diet (23 kJ/kg) for 20 weeks	Treadmill running: HIIT training consisted in 10 bouts at 85%–90% VO_2max_ for 4 min, active rest for 2 min	3 weeks 5 sessions/week	MVO_2_↓, Post-ischemic LV functional recovery↑, infarct size↓	Boardman et al. (2019)
	Oxygen sparing	Mice (C57BL/6J)	71	Male (age, 5–6 weeks) High-fat diet (60% fat) for 20 weeks Ischemic insult	Treadmill running: HIIT training consisted in 10 bouts at 85%–90% VO_2max_ at 25^o^ inclination for 4 min, active rest for 2 min	10 weeks 5 sessions/week	MVO_2_↓, HOMA-IR↓, PFFA↓, systolic and diastolic function↑, infarct size↓, VO_2max_↑	Boardman et al. (2017)
	Oxygen sparing	Mice (C57BL/6J)	38	Male (age, 5–6 weeks) High-fat diet (46% fat) for 8 weeks Ischemic insult	Treadmill running: HIIT training consisted in 10 bouts at 85%–90% VO_2max_ in 25^o^ inclination for 4 min, active rest for 2 min	10 weeks 5 sessions/week	MVO_2_↓, ROS content↓, infarct size↓, diastolic function↑, VO_2max_↑	Lund et al. (2015)
	Cardiomyocyte apoptosis	Rat (Wistar)	48	Male (age,3 weeks) High-calorie diet (20% fat, 68% carbohydrates) for 6 weeks	Treadmill running: HIIT training consisted in 5–16 bouts at 85%–90% MAS for 2 min, active rest at 45%–50% MAS for 2min in 15^o^ inclination	12 weeks 5 sessions/week	Bid↓, cytochrome c↓, caspase-8↓, caspase-3↓, Bax/Bcl-2 ratio↓, Bcl-2 ↑	Astani et al. (2022)
	ER stress	Rat (SD)	30	Male (age, ∼50 weeks) High-fat diet (50% fat) for 6 weeks	Ladder-climbing: HIIT training consisted in 8 repetitions of 80%–100% body weight added at 75^o^ inclination, 2min resting between sets	12 weeks 3 sessions/week	ER stress (p-PERK, CHOP)↓, PGC-1α↓	Kim et al. (2018)
	ER stress	Rat (SD)	40	Male (age, ∼50 weeks) High-fat diet (50% fat) for 6 weeks	Ladder-climbing: HIIT training consisted in 8 repetitions of 80%–100% body weight added in 75^o^ inclination, 2min resting between sets	8 weeks 3 sessions/week	ER stress (p-PERK, CHOP)↓, mitochondrial biogenesis enzymes↑	Kim et al. (2017)
Inflammation	Treg cells	Human	7	MWO BMI = 33.24 ± 2.94 kg/m^2^ (age, 20–40 years)	Treadmill running: HIIT training consisted in 10 bouts of 60s 85%–90% HR_max_ alternated with 75s of recovery 50% HR_max_	1 week 3 sessions/week	Treg and mTreg cells↑	Dorneles et al. (2019)
	Leptin, IFN-γ, IL-4	Human	10	MWO BMI = 35.9 ± 4.9 kg/m^2^ (age, 28.5 ± 2.7 years)	Treadmill running: HIIT training consisted in 10 repetitions at 90% HR_max_ for 1min, active recovery at 30% HR_max_ for 1min	7 sessions	Leptin↓, IFN-γ↓, IFN-γ/IL-4 ratio↓	de Souza et al. (2018)
	Leptin	Human	47	WWO (age, 14.2 ± 1.2 years)	Treadmill running: HIIT training consisted in 3 sessions of 4–8 min of 100% MAS for 15s, active rest at 50% MAS for 15s, passive recovery for 3min between sessions	12 weeks 3 sessions/week	BF↓, WC↓, Plasma leptin↓, HOMA-IR↓, VO_2max_↑, RPE↑, blood insulin↓	Racil et al. (2016a)
	CRP	Rat (SD)	36	Female (age, 18 months)	Treadmill running: HIIT training consisted in 9 bouts of 90%–95% VO_2max_ for 1 min, followed by 40%–45%VO_2max_ for 4 min	32 weeks 5 sessions/week	hsCRP↓, IL-10↑	Sun et al. (2020)
	CRP	Human	74	WWO BMI>30 kg/m^2^ (age, 18–23 years)	Cycling: HIIT training consisted of 5–6 repetitions at 80%–95% HR_max_ for 4–6 min, active recovery at 80%–95% HR_max_ for 2–4 min	12 weeks 3 sessions/week	CRP↓, TC↓, HDL-C↑	Ratajczak et al. (2020)
	CRP	Human	45	MWO BMI = 25.12 ± 1.3 kg/m^2^ (age, 11.06 ± 1.0 years)	Running: HIIT consisted of 3 sets of 10 repetitions at 100%–110% MAS for 10s, active rest at 50% MAS for 10s	12 weeks 3 sessions/week	CRP↓, TC↓, HDL-C↑	Paahoo et al. (2021)
	CRP	Human	38	WWO BMI Z-score = 6,53 ± 2.30 (age, 16.04 ± 1.0 years)	Running: HIIT consisted of 2 blocks of 6 bouts at 100%–110% MAS for 30s, active rest at 50% MAS for 30s	12 weeks 3 sessions/week	CRP↓, BF↓, WC↓, SBP↓	Abassi et al. (2021)
	CRP	Human	39	WWO BMI = 33.0 ± 3.1 kg/m^2^ (age, 15.8 ± 1.6 years)	Treadmill running: HIIT training consisted in 4 bouts of 85%–90% HR_max_ for 4min, active recovery at 70% HR_max_ for 3min	12 weeks 2 sessions/week	hsCRP↓, WC↓, DBP↓	Plavsic et al. (2020)
	CRP	Human	104	PWO BMI = 37.8 ± 6.6 kg/m^2^ (age, 53.7 ± 11.4 years)	Cycling: HIIT training consisted in 5 bouts of 80%–95% HR_max_ for 1min, active recovery at low intensity for 1min	12 weeks 2 sessions/week	CRP↓, hsCRP↓, IL-6↓, LBP↓, MetS↓, SBP↓, DBP↓, VO_2max_↑	Reljic et al. (2022)
	TLR4	Human	38	PWO BMI>24 kg/m^2^ (age, 30–65 years)	Cycling: HIIT training consisted of 4–10 repetitions at 85%–90% HR_max_ for 1min, active rest at 20% HR_max_ for 1min	2 weeks 5 sessions/week	TLR4↓	Robinson et al. (2015)
	Adiponectin	Human	34	WWO BMI = 30.8 ± 1.6 kg/m^2^ (age, 15.9 ± 0.3 years)	Outside running: HIIT training consisted in 2 bouts of 6–8 repetitions of 100% HR_max_ for 30s, active rest at 50% HR_max_ for 30s, recovered passively 4min between series	12 weeks 3 sessions/week	Adiponectin↑, HDL-C↑, VO_2max_↑	Racil et al. (2013)
	Adiponectin	Mice	72	Male (10 weeks old) High-fat diet (45% fat) for 10 weeks	Treadmill running: HIIT training consisted in 8 bouts at 90% MRC for 2 min, active rest at 50% MRC for 2 min	10 weeks 3 sessions/week	HMW adiponectin↑, LMW adiponectin↓, GLUT↑	Martinez-Huenchullan et al. (2020)
	Adiponectin, leptin	Human	68	WWO (age, 16.6 ± 1.3 years)	Treadmill running: HIIT training consisted in 2 bouts of 6–8 repetitions of 100% VO_2max_ for 30s, active rest at 50% VO_2max_ for 30s, recovered passively 4min between series	12 weeks 3 sessions/week	Adiponectin↑, leptin↓, leptin/adiponectin ratio↑, HOMA-IR↓	Racil et al. (2016b)
	Adiponectin, leptin	Human	12	WWO BMI = 29.2 ± 2.6 kg/m^2^ (age, 21.7 ± 3.8 years)	Cycling: HIIT training consisted of 4–6 repeats of Wingate anaerobic test at supramaximal for 30s, interval against a resistance load of 0.065 kg/kg body mass	3 weeks 2 sessions/week	Adiponectin↑, leptin↓	Vardar et al. (2018)
	Adiponectin, leptin	Human	28	PWO (21% male) BMI = 33.0 ± 5.4 kg/m^2^ (age, 61 ± 8.4 years)	Cycling: HIIT training consisted in alternating bouts at 90% HRmax, active rest at 50% HRmax, 60min/session	2 weeks 6 sessions/week	Adiponectin↑, leptin↓, SBP↓, DBP↓, TG↓	Heiston et al. (2020)
	IL-10, TNF-α	Human	33	PWO (age, 30–65 years)	Cycling: HIIT training consisted of 4–10 bouts at 90% HR_max_ for 1min, recovery period for 1min	2 weeks 5 sessions/week	IL-10↑, TNF-α↓	Barry et al. (2018)
	IL-10, IL-8	Human	22	MWO BMI = 25–34.9 kg/m^2^ (age, 20–40 years)	Treadmill running and cycling: HIIT training consisted in 10 bouts of 85%–90% P_max_ for 60s, active recovery at 50% P_max_ for 75s	7 sessions	IL-10↑, IL-8↓	Dorneles et al. (2016)
	HOMA-IR	Human	42	MWO BMI = 26.3 ± 2.1 kg/m^2^ (age, 42 ± 5 years)	Running: HIIT regime consisted of 12 bouts at 85%–90% HRmax for 1min, active recovery at 50% HRmax for 1min	16 weeks 3 sessions/week	HOMA-IR↓, FI↓, VO_2max_↑	Poon et al. (2022)
Insulin resistance	HOMA-IR	Human	18	WWO BMI>30 kg/m^2^ (age, 41–60 years)	Cycling: HIIT regime consisted in 3–4 bouts at 80%–100% HRmax, interval without movement	12 weeks 3 sessions/week	HOMA-IR↓, FI↓, VO_2max_↑	Ratajczak et al. (2020)
	HOMA-IR	Human	42	WWO BMI = 26.3 ± 2.5 kg/m^2^ (age = 21.2 ± 1.4 years)	Cycling: HIIT regime consisted in 9 bouts at 90% V_max_ for 4min, active recovery for 3min, target work 300J/per session	12 weeks 3 sessions/week	HOMA-IR↓, FI↓, VO_2max_↑	Sun et al. (2019)
	HOMA-IR	Human	136	PWO (52.9% males) BMI>27 kg/m^2^ (age, 36 ± 9 years)	Cycling: HIIT regime consisted in 5 bouts at 100%–125% VO_2max_ for 1min, recovery for 1min	6 weeks 3 sessions/week	HOMA-IR↓, MAP↓, VO_2max_↑	Phillips et al. (2017)
	HOMA-IR	Human	73	WWO with insulin resistant (age, 33.5 ± 6.5years)	Cycling: HIIT regime consisted in 8–10 bouts at 70%–100% HRmax, interval without movement	12 weeks 3 sessions/week	HOMA-IR↓, FPG↓, FI ↓, SBP↓, DBP↓	Alvarez et al. (2017)
	OGTT glucose, OGTT insulin	Human	24	WWO BMI>27 kg/m^2^ (age, 18–30 years)	Cycling combined with caffeine: HIIT regime consisted in 10 bouts at 85%–95% HRmax for 60s, dynamic recovery for 60s	8 weeks 3 sessions/week	OGTT glucose↓, VO_2max_↑	Alkhatib et al. (2020)
	Blood glucose	Human	22	WWO with insulin resistant BMI>23 kg/m^2^ (age, 18–30 years)	Cycling: HIIT regime consisted in 60 bouts at ∼90% VO_2max_ for 8s, interspersed with 12s recovery	5 weeks 4 sessions/week	FPG↓, VO_2max_↑	Kong et al. (2016a)
	AFABP	Human	28	PWO (age, 13–18 years)	Running: HIIT regime consisted in alternating bouts at 85%–90% HRmax, active rest at 50%–60% HRmax, 60min/session	6 months 2 sessions/week	AFABP↓	Bluher et al. (2017)
	Myocardial glucose oxidation	Mice (C57BL/6)	40	Male	Treadmill running: HIIT regime consisted in 10 bouts at 85%–90% VO_2max_ for 4 min, active rest for 2 min	8 weeks 5 sessions/week	Myocardial glucose oxidation rate↑, FPG↓, AUG↓, VO_2max_↑	Hafstad et al. (2013)
	Hyperlipidemia	Rat (SD)	30	Male (age, 8 weeks) High-fat diet (58% fat), for 4 weeks	Treadmill running: HIIT regime consisted in 5–10 bouts at 90%–100% VO_2max_ for 2 min, active rest at 50% −60%VO_2max_ for 2 min	8 weeks 5 sessions/week	LDL-C level↓, triglyceride levels↓, HDL↑ ABCs (ABCA1, ABCG1, ABCG4, ABCG8) gene↑, PPARγ↑	Rahmati-Ahmadabad et al. (2019b)
Lipid profile	Blood lipid	Human	24	WWO BMI = 25.8 ± 2.3 kg/m^2^ (age = 21.2 ± 1.4 years)	Cycling: HIIT regime consisted in 60 bouts at 100% VO_2max_ for 8s, interspersed with 12s recovery	5 weeks 4 sessions/week	TC/HDL-C↓, TG/HDL-C↓, VO_2max_↑	Kong et al. (2017)
	NAFLD	Human	65	PWO BMI = 40.9 ± 7.8 kg/m^2^ (age, 52.1 ± 9.6 years)	Cycling: HIIT training consisted in 5 bouts of 80%–95% HR_max_ for 1min, active recovery at low intensity for 1min	12 weeks 2 sessions/week	ALT↓, HbA_1c_↓, NFS↓, MetS↓, SBP↓, DBP↓, MAP↓, VO_2max_↑	Reljic et al. (2021a)
	RAS system	Mice (C57BL/6)	60	Male (age, ∼51 years) High-fat diet (40% fat), High-fructose diet (50% fructose) for 8 weeks, respectively	Treadmill running: HIIT regime consisted at 90% VO_2_ (45 m/min) for 2 min, active rest at 30% VO_2_ (15 m/min) for 1 min	12 weeks 3 sessions/week (alternating)	VAT/SAT↓, LV mass ↓, LV thickness ↓, renin↓, ACE2 mRNA and protein expression↓, Mas receptor ↑, insulin sensitivity ↑	de Oliveira Sa et al. (2017)
Haemodynamics	BP	Human	27	PWO (age, 30–50 years)	Running: HIIT regime consisted in 7–10 bouts at 85%–90% VO_2max_, interval rest for 1 min	8 weeks 3 sessions/week	SBP↓, FPG↓, HOMA-IR↓, BF↓, VAT↓, TC↓, TG↓, VO_2max_↑	Gripp et al. (2021)
	Ventricular function	Rat (Wistar)	46	Male (age, 6 weeks) High-fat diet (23 kJ/kg) for 20 weeks	Treadmill running: HIIT regime at 80% VO_2max_ for 4 min, active rest at 60% VO_2max_ for 3 min	1 week 4 sessions/week	HIIT protocol maintained both myocardial contractility and relaxation after the cardiac insult	Franca et al. (2020)
Cardiac structure and function	Aerobic ability	Human	56	PWO BMI = 36.7 ± 5.0 kg/m^2^ (age = 50 ± 6 years)	Cycling: HIIT consisted of 5 bouts of 95% HR_max_ for 30s intervals with steady state for 2min	12 months 3 sessions/week	LV mass↑, LVEDV↑, AIx↓, VO_2max_ ↑	Hearon et al. (2022)
	Aerobic ability	Human	45	MWO BMI = 24.2 ± 1.0 kg/m^2^ (age = 11.2 ± 0.7 years)	Running: HIIT consisted of 2 sets of 8 repetitions at 90%–100% MAS for 15s, active rest at 50% MAS for 15s	12 weeks 3 sessions/week	VO_2max_ ↑, LDL↓	Diniz et al. (2022)
	Aerobic ability	Human	40	PWO (50% males) BMI = 24.2 ± 1.0 kg/m^2^ (age = 11.0 ± 0.6 years)	Running: HIIT consisted of 3 sets of 8 repetitions at 100% MAS for 15s, active rest at 50% MAS for 15s	12 weeks 3 sessions/week	VO_2max_ ↑	Cao et al. (2022)
	Aerobic ability	Human	60	PWO (81% males) BMI = 31.5 ± 2.8 kg/m^2^ (age = 54.0 ± 11 years)	Cycling: HIIT training consisted in 22 bouts of 100% P_max_ for 60s, passive recovery at 50% P_max_ for 60s	8 weeks 3 sessions/week	VO_2max_ ↑	Mendelson et al. (2022)
	Aerobic ability	Human	31	WWO BMI>23 kg/m^2^ (age, 18–30 years)	Cycling: HIIT regime consisted in 60 repetitions of 80% ± 7% VO_2max_ for 8 s, passive rest for 12 s, 20min/per session	5 weeks 4 sessions/week	VO_2max_ ↑	Kong et al. (2016b)
	Aerobic ability	Human	68	PWO BMI = 28.9 ± 2.9 kg/m^2^ (age, 67.7 ± 4.6 years)	Elliptical cross-training: HIIT training consisted in 10 bouts of 80%–85% HR_max_ for 30s, active recovery at 50%–60% HR_max_ for 90s	12 weeks 3 sessions/week	TG↓, 6MWT↑	Youssef et al. (2022)

Note. 6MWT, 6min walking test; AIx, augmentation index; ALT, alanine aminotransferase; AT, adipose tissue; Bax, Bcl-2-associated X protein; Bcl-2, B-cell lymphoma-2; BF, body fat; Bid, BH3-interacting domain death agonist; BMI, body mass index; BFP, body fat percentage; CHOP, C/EBP homologous protein; CRP, C-reactive protein; DBP, diastolic blood pressure; DGT, defective glucose tolerance; EAT, epicardial adipose tissue; EF, ejection fraction; ER, endoplasmic reticulum; FPG, fasting plasma glucose; FI, fasting insulin; FS, fractional shortening; FAD, flow-mediated dilation; HbA_1c_, glycosylated hemoglobin A_1c_; HDL, high-density lipoprotein cholesterol; HIIT, high intensity interval training; HOMA-IR, homeostatic model assessment of insulin resistance; HRR, heart-rate reserve; hsCRP, hypersensitive serum C-reactive protein; LBM, lean body mass; LBP, lipopolysaccharide-binding protein; LDL, low-density lipoprotein cholesterol; LV, left ventricular; LVEDV, LV end-diastolic volume; MAP, mean arterial pressure; MAS, maximal aerobic speed; MetS, metabolic syndrome; MRC, maximal running capacity; MVO_2_, myocardial oxygen consumption; MWO, men with obesity; NAFLD, Non-alcoholic fatty liver disease; NFS, Non-alcoholic fatty liver disease fibrosis score; PAT, paracardial adipose tissue; P_max_, maximum power output; p-PERK, phosphor-protein kinase RNA-like endoplasmic reticulum kinase; PFFA, plasma free fatty acid; PWO, people with obesity; RPE, ratings of perceived exertion; SAT, subcutaneous adipose tissue; SBP, systolic blood pressure; SD, Sprague-Dawley; TC, total cholesterol; TG, triglycerides; Treg cells, regulatory T cells; mTreg cells, memory regulatory T cells; VAT, visceral adipose tissue; WC, waist circumference; WWO, women with obesity; "↑", increase; "↓", decrease.

### 5.1 HIIT regulates the heart adipose tissue distribution

The AT in the human body is classified as white AT (WAT) and brown AT (BAT) morphologically, and subcutaneous AT (SAT) and visceral AT (VAT) anatomically (Britton et al., 2013). Excessive caloric intake results in an enlargement of the AT via the number of adipocytes (hyperplasia) and/or size (hypertrophy). Under the physiological context of energy balance, lipids should be stored in the insulin-sensitive SAT that expands through hyperplasia to avoid the overflow of excess lipids to other organs (Koliaki et al., 2019). However, anomalies in SAT deposition will likely lead to excess triglycerides being directed to ectopic sites like the liver, heart, and skeletal muscle (Koliaki et al., 2019). Ectopic AT is characterized by hypertrophic expansion from pre-existing adipocytes. VAT, fat stored around internal organs, can be defined according to its anatomical position as intrathoracic or abdominal, and intrathoracic AT can be further classified as epicardial adipose tissue (EAT) and pericardial adipose tissue (PAT). EAT is anatomically and functionally continuous with the myocardium, whereas PAT is located between the visceral and parietal pericardium (Rafeh et al., 2020; Despres et al., 2021). The accumulation of excess VAT and PAT plays independent and crucial roles in the pathophysiology of cardiac structural and functional changes (Kenchaiah et al., 2021; Kim et al., 2021; Kondamudi et al., 2021; Sorimachi et al., 2021).

A number of studies suggest that HIIT might be an efficient VAT and EAT reduction strategy (Nyawo et al., 2021; Saco-Ledo et al., 2021). For example, 3 months of a HIIT cycling program (90% VO_2_max, 3-4 sessions/week) decreased the VAT area (−50.2 cm^2^) (Tong et al., 2018) and raised VO_2_max (Zhang et al., 2017) in young women (aged 18–23 years) with obesity. EAT is a typical maker of VAT that exerts detrimental effects on the myocardial architecture through an elevated LV mass, a deranged RV geometry, susceptible arrhythmogenicity, and increased lipotoxicity (Iacobellis, 2015; Le Jemtel et al., 2019; Neeland et al., 2019; Nalliah et al., 2020). EAT is a significant source of cardiac M1 macrophages (Mouton et al., 2020), a local transducer of systemic inflammation to the myocardium, and a provenience of pro-inflammatory adipocytokines, such as tumor necrosis factor-α (TNF-α) and IL-6 that facilitate the generation of an inflammatory microenvironment. These factors contribute to myocardial disarray, fibrosis, and stiffness in uncorrected obesity (Packer, 2018; Le Jemtel et al., 2019; Rafeh et al., 2020; Wu and Ballantyne, 2020). After 12 weeks of high-intensity endurance training cycling (75%–85% VO_2_max, 3 sessions/week), EAT was reduced by 32% in previously sedentary adults (mean age 41 years, 26% male) with abdominal obesity due to the isolated effect of HIIT without dietary restrictions (Christensen et al., 2019). Eight weeks of high-intensity interval running (75%–80% heart-rate reserve, 3 sessions/week) could be more effective than MICT to reduce the EAT thickness and improved endothelial function in hypertensive adults (mean age 50.9 years, 51% male) (Jo et al., 2020). Only 2 weeks of maximal all-out cycling (3 sessions/week) resulted in a significant decrease in EAT and PAT, along with increased aerobic capacity and insulin sensitivity in men (aged 43–53 years) affected by obesity with defective glucose tolerance (DGT), whereas HIIT appears superior to MICT (Honkala et al., 2017). Thus, the findings of these studies provide insights for HIIT for the regulation of the adipocyte distribution, especially EAT.

Despite the many prospects of HIIT, the paucity of studies on the effect of HIIT on the PAT distribution merits more investigation. Additionally, there exist limitations in the abovementioned studies due to the heterogeneity of the participant characteristics or the HIIT protocol. As a result, future research might include the detection of additional biochemical and molecular indicators and an evaluation of HIIT in the AT distribution correlated with cardiac impairment in individuals with obesity or overweight.

### 5.2 HIIT modifies the adipose tissue microenvironment in obesity

Under a hypercaloric or over nourished state, the AT can multiply through two unique processes, hyperplasia/adipogenesis (the differentiation of new adipocytes from progenitor cells) and hypertrophy (an increase in the adipocyte size). AT hyperplasia is considered a healthy mechanism for forming new functional adipocytes from the mobilization of preadipocytes without altering their secretory profile while preserving the AT vascularization milieu (Vishvanath and Gupta, 2019). Different from hyperplasia, the hypertrophy and subsequent adipocyte dysfunction lead to a hypoxic state on account of the imbalance between the expanding AT mass with increased oxygen consumption and the decreased capillary density for less oxygen delivery (Smith et al., 2019). Hypoxic conditions (intra-adipocyte oxygen concentration <1.4%) lead to greater expression of the adipocyte hypoxia-inducible factor-1α, nuclear factor kappa B (NF-κB), and cAMP response element-binding protein genes, and all of these are related to oxidative stress activation and pro-inflammatory transcriptome response to induce myocardium damage (Trayhurn, 2013; Bermudez et al., 2021).

As mentioned above, a hallmark of obesity-related cardiac damage is an imbalance between the increased oxygen consumption of cardiac activity and the decreased oxygen availability during the AT expansion process. When the oxygen supply is restricted, a high myocardial oxygen consumption (MVO_2_) is particularly detrimental to rendering the myocardium more susceptible to ischemia injury. Increased fatty acid supply and/or utilization, impaired mitochondrial function and oxidative stress, and altered Ca^2+^ handling all contribute to pathological alterations in ATP synthesis and/or utilization in an obese heart. Compared to MICT, 3 months of high-intensity interval walking (75%–85% VO_2_max, 3 sessions/week) could significantly upregulate the fat oxidation rate and ADP-stimulated mitochondrial respiration to improve mitochondrial function in adults with obesity (mean age 39 years, 53% males) (Vaccari et al., 2020). In addition, HIIT exerts a cardioprotective effect through a myocardium oxygen sparing mechanism in high-fat load rodents. Ten weeks of HIIT (running, 85%–90% VO_2_max, 25^o^ inclination, 5 sessions/week) improved the postischemic cardiac functional recovery, aerobic capacity, and glucose tolerance by decreasing MVO_2_ in male mice (5–6 weeks old) with obesity (Boardman et al., 2017). Another comparable study demonstrated that HIIT (running, 85%–90% VO_2_max, 25^o^ inclination, 5 sessions/week) reduced the amount of myocardial reactive oxygen species (ROS) and fibrosis in the myocardium, reducing the mechanical efficiency degradation caused by obesity in male mice (5–6 weeks old) (Lund et al., 2015). Even with a three-week training period, HIIT (running, 85%–90% VO_2_max, 25^o^ inclination, 5 sessions/week) was able to decrease myocardial oxygen wasting and increase oxidative phosphorylation, ameliorating post-ischemic LV diastolic dysfunction and reducing infarct size in mice affected by obesity (Boardman et al., 2019). Additionally, obesity triggers cardiomyocytes to undergo apoptosis by enhancing mitochondrial permeability, cytochrome c release, caspase-8 and caspase-3 upregulation in cardiomyocytes. Twelve weeks of HIIT (running, 85%–90% maximum aerobic speed, 5 sessions/week) effectively reduced pro-apoptotic proteins, including cytochrome c release, caspase-8, and caspase-3 to suppress cardiomyopathy and HF in rats (3 weeks old) with obesity (Astani et al., 2022).

Moreover, increased plasma free fatty acids (FFAs) increased endoplasmic reticulum stress (ERS), and this occurred concurrently with increased ROS generation of the mitochondria. The protein kinase RNA-like ER kinase (PERK) is a pivotal branch of unfolded protein response (UPR) in mitochondria malfunction associated with ER stress during obesity development. Eight weeks of high-intensity ladder climbing (80%–100% body weight added in 25^o^ inclination, 3 sessions/week) was shown to decrease the levels of ER stress-related proteins, such as the phosphor-PERK (p-PERK)/PERK ratio and the C/EBP homologous protein (CHOP), a downstream of PERK that led to myocardium injury in male rats (50 weeks old) with obesity (Kim et al., 2017; Kim et al., 2018). This type of intervention also increased the peroxisome proliferator-activated receptor δ (PPARδ) and mitochondrial enzyme levels to reduce ER stress and improve mitochondrial efficiency through a decreased PERK/CHOP pathway (Kim et al., 2017). PPARδ is a nuclear receptor that is activated by FFAs, as well as an efficient activator in reducing ROS-associated pathway activity in the myocardium. Thus, the HIIT induced improvement in cardiac function partly due to the regulation of myocardial oxygen metabolism, mitochondrial respiration, and ERS under a hyperlipidemic condition.

As a matter of fact, the period of HIIT intervention was limited to 12 weeks, and the study subjects related to HIIT in obesity-induced mitochondrial impairment and ERS focus on rodents, which did not provide sufficient data for the application and efficacy of HIIT in the adipose microenvironment of the myocardium caused by obesity in clinical patients.

### 5.3 HIIT alleviates the inflammatory reaction in an obese heart

The development of a hypoxic microenvironment during abnormal AT expansion plays a predominant role in activating adipocytes and resident immune cells, releasing a flood of secretory factors termed adipokines (Guzik et al., 2017; Leake, 2019; Seo et al., 2019). Macrophages have been identified as the major cells of this system in AT. In individuals with obesity, the hypoxic state of AT results in dysregulation of the adipokines, including interferon-γ (IF-γ) by T helper 1 (Th1) lymphocytes, to promote anti-inflammatory M2 macrophage transformation to the pro-inflammatory M1 phenotype (primarily derived from EAT) recruitment and polarization. This results in an increased release of pro-inflammatory cytokines, such as leptin, transforming growth factor-β, nitric oxide, TNF-α, interleukin (IL)-6, C-reactive protein (CRP), and toll-like receptors (TLRs), and a decreased secretion of adiponectin, fibroblast growth factor 21, IL-10, and IL-33 (Guzik et al., 2017; Lee and Olefsky, 2021). In this scenario, the abnormal accumulation of AT upregulates pro-inflammatory adipokines and downregulates anti-inflammatory ones, and this contributes to triggering the development of a chronic local and systemic inflammatory reaction, IR, RAAS activation, and lipotoxicity, all of which may act synergistically with adipokines in the onset and progression of cardiac damage (Larabee et al., 2020; Ren et al., 2021).

#### 5.3.1 Regulation of cellular immune dynamics

Macrophages are present in less than 10% of AT in lean mice and humans but can reach up to 40%–50% in individuals with obesity and leptin-deficient rodents with obesity by local proliferation and infiltration (Amano et al., 2014). Furthermore, resident macrophages occur in low numbers in a healthy heart, where they usually adopt an M2 phenotype but can become inflammatory with obesity. Under the obese context, macrophages promote pathological hypertrophy and impair systolic and diastolic function in the heart via pro-inflammatory cytokines (TNF-α, IL-1β, IL-6) that stimulate cascade M1 macrophages that are responsible for the inflammation associated with the pathological cardiac injury in obesity (Hulsmans et al., 2018; Mouton et al., 2020).

HIIT is a superior exercise approach for boosting capillary and anti-inflammatory macrophages in the AT of male rats with obesity and normal weight, therefore ameliorating inflammatory responses and IR (Kolahdouzi et al., 2019). Relatively high-intensity training is more efficient for M2 macrophages polarization in AT when compared with the same total exercise amount in male mice (Baek et al., 2020). Just three sessions of HIIT (running, 85%–90% HRmax) reverted AT inflammation by increasing the frequencies of regulatory T (Treg) cells, whose primary job is to maintain immunological homeostasis in men (aged 20–40 years) with obesity. This was followed by an increase in anti-inflammatory cytokines (IL-10, IL-33) (Dorneles et al., 2019). These findings revealed that HIIT is an effective method to switch macrophage polarization to the M2 phenotype and modulate numerous features of immune cells during AT accumulation, but further studies are required to decipher the macrophage-polarization process and how this regulates the mechanisms of exercise intervention in obesity.

#### 5.3.2 Decreased pro-inflammatory adipokines

Leptin is a 16-kDa peptide hormone that is primarily released by adipocytes. It is an adipokine that can modulate the activity of the pro-inflammatory phenotype Th1 and the sympathetic nervous system in individuals with obesity (Caron et al., 2018). Th1 lymphocytes generate IFN-γ, an essential inducer of macrophage polarization to a pro-inflammatory phenotype (M1), and they also contribute to systemic inflammation by producing inflammatory cytokines such as tumor necrosis factor-alpha (TNF-α). Leptin level is increased in obesity to correspond with cardiac hypertrophy by binding of leptin to the short form of the leptin receptor in rat hearts (Rajapurohitam et al., 2003; Zhao et al., 2021). Short-term high-intensity interval running (90% HRmax, 7 sessions) had a greater impact than MICT in decreasing the leptin levels and the IFN-γ expression in males with obesity (mean age 28.5 years). The reduction in IFN-γ by HIIT may signify a transient reduction in T1-mediated immunity (de Souza et al., 2018). Moreover, 12 weeks of HIIT (running, 100% maximum aerobic speed, 3 sessions/week) decreased the blood leptin concentration and body fat mass, resulting from increased VO_2_max at post-intervention in young women with obesity. It revealed that leptin sensitivity might revert to normal functioning when adipose tissue was decreased and HIIT-induced aerobic fitness (VO_2_max) promotion in female teenagers (mean age 14.2 years) affected by obesity (Racil et al., 2016a).

C-reactive protein (CPR) is a pentraxin protein primarily synthesized and secreted by the liver and released into the bloodstream in response to inflammation in obesity (Yang et al., 2021). Reduced levels of CRP have been linked to an improvement in cardiorespiratory fitness. Eight months of high-speed interval running (90%–95% VO_2_max, 5 sessions/week) reduced the CRP levels in aged female rats (18 months) (Sun et al., 2020). Twelve weeks of high-intensity indoor cycling (80%–95% HRmax, 3 sessions/week) was an effective stimulus for improving the aerobic capacity and reduce the levels of the CRP in women (aged 40–60 years) with obesity (Ratajczak et al., 2020). Among boys (mean age 11.06 years) (Paahoo et al., 2021), girls (mean age 16 years) (Plavsic et al., 2020; Abassi et al., 2021), and women (aged 40–50 years) affected by obesity (Ratajczak et al., 2020), 3 months of HIIT was more effective than aerobic training for decreasing CRP and related cardiovascular risk factors. In addition, 3 months of low-volume high-intensity interval cycling (80%–95% HRmax, 2 sessions/week) meaningfully reduced CRP and IL-6 while increasing VO_2_max in metabolic syndrome patients (mean age 53.7 years) with obesity (Reljic et al., 2022). However, it has been noted that CRP was elevated after 3 weeks of HIIT (84%–87% HRmax, 4 sessions/week) and reduced in MICT in young adults (aged 18–44 years, 41% male). This phenomenon was partially related to inflammation induced by short-term exercise (Vella et al., 2017).

Toll-like receptors (TLRs), are crucial in early innate immunity because they recognize both pathogen-associated and endogenous damage-associated molecular patterns. Increased TLR2 and TLR4 expressions, as well as the pro-inflammatory milieu that results, are related with a variety of cardiometabolic risk factors, including IR, type 2 diabetes, and atherosclerosis (Ji et al., 2019). Similar to MICT, 2 weeks of HIIT (cycling, 85%–90% HRmax, 5 sessions/week) reduced the TLR2 and TLR4 expressions to improve glucose control and cardiorespiratory fitness in inactive adults (mean age 52 years, 15% male) with obesity (Robinson et al., 2015). This research established that a decrease in TLRs on immune cells may be a direct anti-inflammatory response to short-term high-intensity exercise training.

#### 5.3.3 Increased anti-inflammatory adipokines

Adiponectin is one of the most abundant adipokines related to anti-inflammatory, insulin sensitization, and lipid metabolism properties (Li et al., 2020; Maeda et al., 2020; Fontanella et al., 2021). Adiponectin occurs as three oligomeric multimers in circulation: a low-molecular-weight trimer, a medium molecular weight hexamer, and a high molecular weight (HMW) multimer. HMW adiponectin represents the biologically most active form of adiponectin (Straub and Scherer, 2019). Additionally, adiponectin prevents cardiomyocyte hypertrophy and myocardial fibrosis in individuals with obesity. A decline in circulating adiponectin levels and a loss of cardioprotective effects occur concurrently with increased body fat mass, particularly visceral fat expansion (Botta et al., 2019; Fontanella et al., 2021). Following 12 weeks of high-intensity interval running (100% HRmax, 3 sessions/week), the plasma adiponectin and high-density lipoprotein-C (HDL-C) concentration increased dramatically in adult women (mean age 15.9 years) affected by obesity (Racil et al., 2013). A similar protocol also was demonstrated to upregulate the adiponectin/leptin ratio in female teenager (mean age 16.6 years) (Racil et al., 2016b). Ten weeks of high-intensity interval running (90% maximum running capacity, 3 sessions/week) increased the myocardial high-molecular adiponectin expression, glucose transporter type 4 (GLUT4) translocation, and glucose uptake after a high-fat diet in ten-week-old male mice (Martinez-Huenchullan et al., 2020). This finding suggested the regulatory function of adiponectin on glucose metabolism and IR in obesity development (Martinez-Huenchullan et al., 2020). In addition, increased adiponectin and decreased leptin have been observed after a short term high-intensity interval cycling training in young female (mean age 21.7 years) (Vardar et al., 2018) and aged adults (mean age 61 years, 21% male) affected by obesity (Heiston et al., 2020). The transient alterations may imply a favorable function of adipokines in increasing anaerobic capacity and lowering the fat mass ratio in females who are overweight/obese (Vardar et al., 2018).

Interleukin 10 (IL-10) is primarily released by Treg cells and plays a critical role in immunomodulation by suppressing various cells such as T and B lymphocytes, macrophages, and dendritic cells. Since obesity reduces IL-10 levels, systemic IL-10 treatment was observed to significantly alleviate LA remodeling and the susceptibility to AF in diet-induced obesity (Kondo et al., 2018). Compared to MICT, short-term HIIT (running, 85%–90% maximum power output, 7 sessions) displayed a more substantial effect on the increased IL-10 level (Dorneles et al., 2019) and a concomitant decrease in the IL-8 concentration, a pro-inflammatory factor released from mononuclear cells (Dorneles et al., 2016) in men (aged 20–40 years) with obesity. Moreover, HIIT-induced IL-10 overexpression may suppress IL-β, IL-6, and TNF-α in mononuclear cells challenged with lipopolysaccharide and the transcription of IL-8 in polymorphonuclear leukocytes in individuals with obesity (Dorneles et al., 2016; Barry et al., 2018). The negative association between IL-10 and IL-8 during HIIT may reaffirm the immunoregulatory role of IL- 10 in the regulation of pro-inflammatory cytokine production and release. Thus, HIIT may have significant consequences for creating anti-inflammatory benefits in patients affected by obesity with chronic low-grade inflammation.

Consequently, many studies have indicated that HIIT has immunomodulatory effects on the obese heart due to decreased pro-inflammation, increased anti-inflammation, and the transition of M1 and M2 immune cells. However, some studies have shown that HIIT had no significant ameliorative effect on obesity-induced inflammatory responses in adults (Mora-Rodriguez et al., 2018; Kao et al., 2021). This might have been due to the participants’ physiological condition and complex variable training factors of the HIIT programs in these studies.

### 5.4 HIIT improves insulin resistance (IR) in the obese heart

Most research supports the notion that increased pro-inflammatory macrophages in AT caused by obesity might directly promote IR. IR is a term that refers to a decrease in the metabolic response to insulin in target cells or, at the whole-organism level, a requirement for higher insulin levels to lower blood glucose (Czech, 2017). The heart is a critical insulin-responsive organ that might develop IR due to obesity (Broussard et al., 2016; Hirose et al., 2021). Notably, the metabolic milieu associated with IR is characterized by elevated circulating glucose, free fatty acids, triglycerides, and a dysregulated substrate supply from the periphery to the heart, increased fatty acid oxidation, and decreased glucose uptake and oxidation, and altered gene expression in cardiomyocytes. These factors contribute directly to the development of adverse cardiac remodeling and dysfunction in obesity (Nakamura and Sadoshima, 2020). According to one study that included 3,179 individuals, severe IR in early adulthood was related with increased LV wall thickness and worse longitudinal systolic strain as well as an increased rate of early diastolic strain in middle age, depending on the severity of obesity (Kishi et al., 2017). Among adult men, the homeostatic model assessment for insulin resistance (HOMA-IR), a critical indicator of insulin sensitivity, accounted for 26% of the total effect on HF risk. While, in women, the HOMA-IR accounted for 29% of the effect on HF risk (Savji et al., 2018). In addition, glucose intolerance displayed a strong correlation with LV mass/wall thickness in subjects with overweight and obesity.

HIIT programs have played a positive role in glycemic control, IR, and cardiovascular function improvements in obesity. For instance, 12 weeks of high-intensity interval cycling (70%–100% HRmax, 3 sessions/week) exerted a reduced in fasting plasma glucose (−6.6%), fasting insulin (−47.2%), HOMA-IR (−50%), and systolic blood pressure (−6 mmHg) in women (mean age 33.5 years) affected by obesity with IR (Alvarez et al., 2017). Compared to MICT, three to 4 months of HIIT exerted apparent effects on fasting insulin decreases, and cardiovascular fitness increases in males (mean age 42 years) (Poon et al., 2022) and females (mean age 21.2 years) (Sun et al., 2019) with obesity. A meta-analysis involving 379 children (aged 7–19 years) with obesity noted that HIIT significantly impacted the cardiometabolic risk factors, especially HOMA-IR, fasting glucose, and fasting insulin (Zhu et al., 2021). Several HIIT-like regimens (90% VO_2_max, 4 sessions/week) have been tested on female patients (mean aged 19.8 years) with obesity to manage their cardiovascular and metabolic health (Kong et al., 2016b). One study applied 6 weeks of “5-by-1″ high-intensity interval cycling (100%–125% VO_2_max, 4 sessions/week) to produce beneficial training responses, including a reduction in the HOMA-IR (−16%) and mean arterial pressure (−3%) and a simultaneous increase in VO_2_max (+10%) in adults (mean age 36 years, 52.9% males) affected by obesity (Phillips et al., 2017). Another remarkable study indicated that, 8 weeks of high-intensity interval cycling (85%–95% HRmax, 3 sessions/week) combined with caffeine intake significantly improved obesity-induced hyperinsulinemia, hyperglycemia, endotoxicity, cardiorespiratory, and anaerobic fitness in women (aged 18–30 years) with obesity. Moreover, synergetic HIIT-caffeine dramatically ameliorated the HIIT-induced side effects of increased endotoxicity and insulinemia in obesity (Alkhatib et al., 2020). In the high-fat induced obese mice, 10 weeks of high-intensity interval running (90% maximal running capacity, 3 sessions/week) elevated myocardial GLUT4 translocation and glucose uptake (Martinez-Huenchullan et al., 2020). Additionally, 8 weeks of high-intensity interval running (85%–90% VO_2_max, 5 sessions/week) dramatically increased the rates of myocardial glucose oxidation in the myocardium of obese mice. This finding proved that HIIT induced a slight alteration in substrate utilization toward an increased in glucose utilization by myocardium (Hafstad et al., 2013). Although direct evidence that HIIT improves IR in obese myocardium is insufficient, HIIT has been shown to improve insulin-stimulated myocardial glucose uptake (MGU) in healthy men. One study indicated that 2 weeks of high-intensity interval cycling significantly decreased the MGU along with whole body increased insulin sensitivity in healthy heart (Eskelinen et al., 2016). In brief, HIIT conveys benefits to improve obesity-induced cardiometabolic risk, and in the instance of IR and VO_2_max, it may be suitable and superior to the effect of traditional continuous training. However, more studies are required to determine the regulatory impacts of HIIT on myocardium IR and related signaling pathways in cardiac damage caused by obesity.

### 5.5 HIIT improves lipid profile related to the obese heart

One of the primary mediators of intracellular transport of fatty acids is adipocyte fatty acid-binding protein (AFABP), a lipid chaperone abundantly produced by adipocytes and macrophages. AFABP interacts with c-Jun NH2-terminal kinase (JNK) and the activator protein-1 to form a positive feedback loop. The overproduction of AFABP leads to cholesterol and triglyceride accumulation, as well as pro-inflammatory factors increase. It is believed to be implicated in inflammatory responses, IR, and metabolic regulation, all of which are associated with increased cardiovascular mortality and inflammation (Lee et al., 2021). After 6 months of HIIT (running, 85%–95% HRmax, 2 sessions/week), AFABP significantly decreased and correlated with fasting insulin, HOMA-IR, and triglycerides in adolescents (mean age 15.5 years) with obesity. This data indicated that AFABP might positively function in glucose homeostasis and metabolic syndrome in teenagers affected by obesity (Bluher et al., 2017).

Atherosclerosis is more likely to occur in individuals with obesity. High-density lipoprotein (HDL) is a predictor of atherosclerosis and a factor in assessing cardiac fitness (Sacks et al., 2020). Apolipoprotein A1 (Apo A-1) may be a more sensitive indicator of cardiac disease than HDL (May et al., 2016). Exercise plays a positive role in lipid and lipoprotein regulation to reverse cholesterol transport in animal study (Rahmati-Ahmadabad et al., 2019a). This process comprises multiple significant components, including adenosine triphosphate-binding cassette transporters (ABCA1, ABCG1, ABCG4, ABCG5, and ABCG8), Apo A-1, and lecithin-cholesterol acyltransferase, all of which are involved in HDL synthesis. This procedure extracted cholesterol from vessels and transported it to the liver for bile extraction. The peroxisome proliferator-activated receptor-gamma (PPARγ) and the liver X receptor-α (LXR-α) affect the ABC transporters to regulate cholesterol effuse (Xia et al., 2012). Eight weeks of high-intensity interval running (90%–100% VO_2_max, 5 sessions/week) stimulated cardiac tissue PPARγ, LXR-α, and ABCs (ABCA1, ABCG1, ABCG4, ABCG8) expression to upregulate the plasma HDL to act against atherosclerosis in male rats (8 weeks old) with obesity (Rahmati-Ahmadabad et al., 2019b). Twelve weeks of high-intensity cycling training significantly increased the HDL-C concentration and decreased the total cholesterol in women (aged 41–60 years) with obesity (Ratajczak et al., 2020). Another study also indicated that 5 weeks of HIIT (cycling, 90%–100% VO_2_max, 4 sessions/week) positively affected bold lipids and cardiorespiratory fitness in young females (aged 18–30 years) with overweight (Kong et al., 2017).

Moreover, non-alcoholic fatty liver disease (NAFLD) and cardiometabolic disturbance are extremely common in individuals who suffer from obesity. Twelve weeks of high-intensity interval cycling (85%–95% HRmax, 2 sessions/week) could dramatically decreased alanine aminotransferase (ALT) and increased VO_2_max in patients (mean age 52.1 years) with obesity and metabolic syndrome (MetS) (Reljic et al., 2021a). This effect might be accomplished by reducing the accumulation and activation of monocyte-derived macrophages (Fredrickson et al., 2021), improvement of adiponectin and leptin, and enhancing the hepatic beta-oxidation (de Castro-de-Paiva et al., 2022) through HIIT intervention in NAFLD. Thus, HIIT yielded the amelioration in the cardiac fitness in individuals with obesity, supporting the positive effect of a HIIT regime for cardiac lipometabolic risk in obesity development.

### 5.6 HIIT regulates hemodynamics in obesity

Excessive carbohydrate and fat intake tend to produce hypertension, RAS dysfunction, and cardiac remodeling to induce a hemodynamic disturbance in obesity. In high-fat diet mice, 12 weeks of HIIT (running, 90% VO_2_max, 3 sessions/week) displayed a remarkable reduction in systolic blood pressure (SBP) (−6.5%, relative to 10 mmHg) and LV mass (−8.5%) compared to the sedentary group (de Oliveira Sa et al., 2017). Moreover, HIIT modulated the expression of LV-RAS axis-related components, including the ACE-Ang II-AT1R and ACE2/Mas receptors (de Oliveira Sa et al., 2017). After 1 month of HIIT, the SBP was significantly decreased in adults (aged 30–50 years) with obesity (Gripp et al., 2021). Three months of HIIT intervention (running, 85%–95% HRmax, 2 sessions/week) accompanied by nutrition advice decreased the systolic and diastolic blood pressure and improved cardiac fitness in adolescent girls (mean age 16 years) with obesity (Plavsic et al., 2020). Thus, HIIT regulates hemodynamics through lowering blood pressure and local RAS system in the heart to ameliorate cardiac damage associated with obesity.

## 6 Brief guidelines for the performance and surveillance of HIIT in individuals with obesity

**FIGURE 3:**
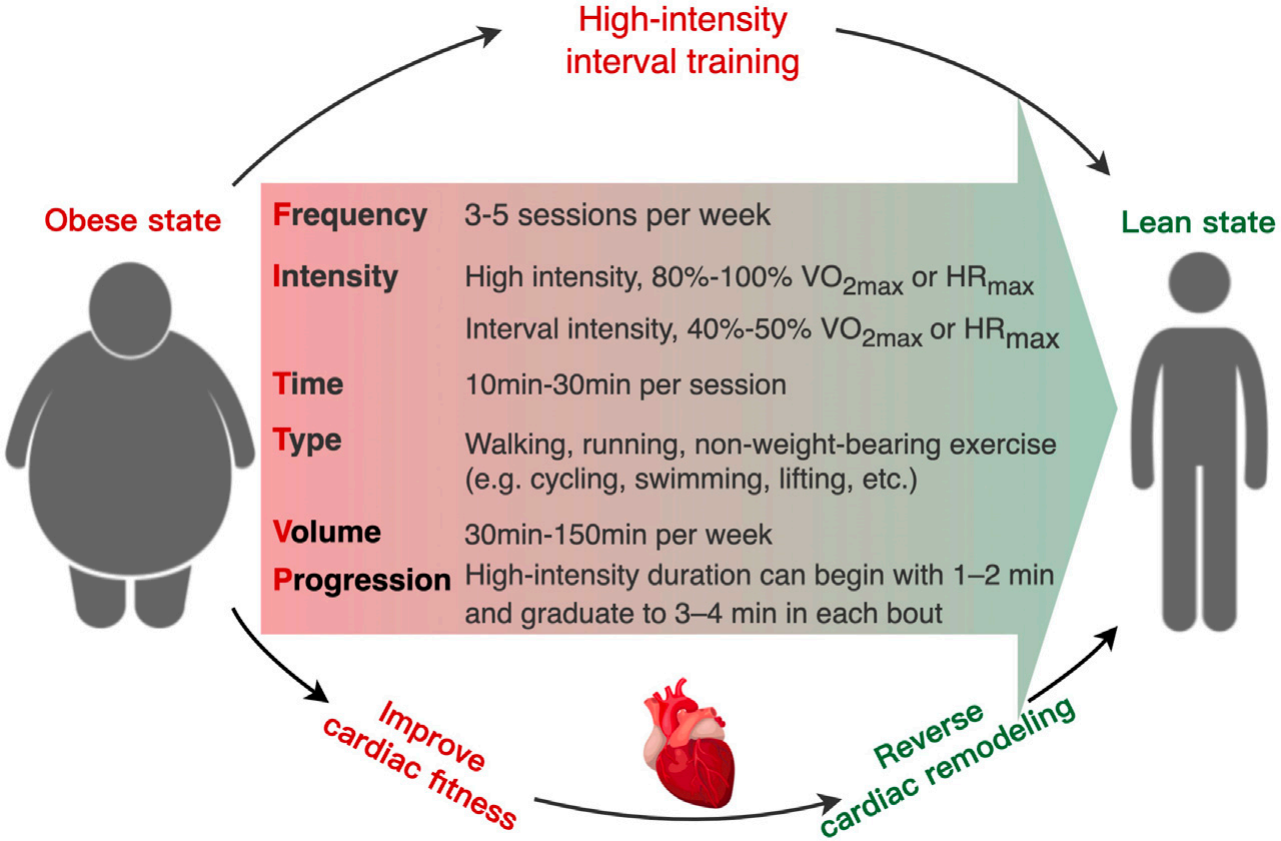
Schematic of the recommended “FITT-VP” framework (frequency, intensity, time, type, volume, and progression) for prescribing high-intensity interval training to obesity-induced cardiac remodeling in individuals with overweight and obesity.

Although HIIT is an effective and low-cost non-pharmacological therapy method, the safety of HIIT in individual with severe obesity and/or cardiac vulnerability should be considered during implementing HIIT as routine therapy (Eckel et al., 2014; Riebe et al., 2018). Currently, a contentious issue arises regarding the potential adverse effects of high-intensity exercise on cardiac health, particularly in certain individuals (La Gerche and Heidbuchel, 2014). There is a scarcity of data on the safety of HIIT in clinical populations with obesity. A systematic analysis revealed that the utilization of HIIT in individuals with cardiometabolic diseases resulted in an adverse event rate of 8% (Levinger et al., 2015). Recent studies indicate that engaging in HIIT may potentially lead to negative cardiovascular consequences, including heightened susceptibility to AF, the development of coronary artery calcification, and the formation of myocardial fibrosis (Mozaffarian et al., 2008; von Klot et al., 2008; Eijsvogels et al., 2016; Franklin et al., 2020). Consequently, professional groups, like as the American Heart Association (AHA) and American College of Sports Medicine (ACSM) have consistently advocated for the utilization of cost-effective screening methods and development of gradually progressive exercise regimens to mitigate the risk associated with HIIT in individual who suffer from obesity or cardiometabolic disease (Guazzi et al., 2012; Stone et al., 2014; Riebe et al., 2018; Arnett et al., 2019). Due to the diversity of HIIT protocols, there are no universal criteria or framework for prescribing and monitoring HIIT in clinical groups (Jensen et al., 2014). The most common uncertainties in prescription and performance of HIIT for individuals with overweight or obesity include the specific exercise intensity and durations of high and low intervals, the method for prescribing exercise intensity (e.g., percentage of peak HR, rating of perceived exertion, etc.), and participant safety (Dun et al., 2019; Taylor et al., 2019). As HIIT generates the near-maximal exercise intensity, the primary prerequisite is to apply appropriate screening (e.g., electrocardiogram, electrocardiography, haemodynamic variables) to assess the suitability and identify risk factors of HIIT participants prior to HIIT intervention (Fletcher et al., 2013; Riebe et al., 2015). To maximize the safety of participants with obesity, a framework for HIIT prescription could incorporate objective and subjective measurements of exercise intensity (HR target zone) (Taylor et al., 2019), including measuring the maximal heart rate (HRmax) through cardiopulmonary exercise testing (Guazzi et al., 2012; Lavie et al., 2018) or predictive equation [e.g., HRmax = 211 − (0.64×age)] (Nes et al., 2013). However, it should be noted that due to significant inter-individual variability, training prescriptions based on such equations may be associated with error. The HR target zone of HIIT is corresponding to 85%–95% HRmax, which could be validated by rating of perceived exertion (RPE). For this purpose, the intensity of the starting workload in HIIT is matching to a participant’s RPE 15 (hard), and the finishing workload at RPE 17–18 (very hard) (Taylor et al., 2019; Andreato, 2020). In addition, participants should be monitored (e.g., average training HR and RPE, peak training HR and RPE, blood pressure) and questioned (e.g., dizziness, palpitation, angina, and dyspnea) regularly following medical clearance to identify any symptoms preclude HIIT implementation (Lavie et al., 2018; Taylor et al., 2019). To guarantee appropriate progression, the intensity target should be increasing the workload (e.g., speed/incline on a treadmill or watts on a bike) on a regular (e.g., weekly) basis (Taylor et al., 2019). For the safety of individuals affected by severe obesity or/and cardiac vulnerability, the warm-up period could be extended from 5–10 min–10 min and the cool-down duration could be increased from 3 min to 3–5 min when conducing HIIT (Taylor et al., 2019). To progressively introduce HIIT, the prescription can begin with shorter interval periods (1–2 min) and graduate to longer interval periods (3–4 min) as exercise intolerance and physical fitness improve in obesity (Taylor et al., 2021). Thus, appropriate pre-exercise screening of contraindication and regular monitoring of physiological responses could maximize the safety and effectiveness when applying HIIT within the obesity setting. Regarding to the exercise type, several studies indicated that non-weight-bearing exercise (such as swimming, rowing, lifting, etc.) with a low risk of injury might enhance the positive effects of HIIT on metabolic health by recruiting multiple lower and upper body muscle groups in individuals who suffer from obesity (Reljic et al., 2019; Petersen et al., 2022). In addition, training volume is a considerable factor in the efficacy of HIIT treatment. A growing body of evidence suggests that a low-volume exercise program can produce comparable or even superior improvements in cardiorespiratory fitness (CRF) than MICT, despite significantly less training volume and time consumption (Ramos et al., 2017; Reljic et al., 2021b; Ramos et al., 2021; McGregor et al., 2023). Reljic et al. demonstrated that only 28min of low-volume HIIT per week could result in dramatically improvement in CRF and cardiometabolic healthy in obesity with MetS (Reljic et al., 2021b). Therefore, safety training intensity, appropriate training type, and effective training volume are the cornerstone in the HIIT therapy in obesity (Figure 3).

## 7 The limitations of HIIT prescribed for obesity

As aforementioned, HIIT has displayed improvements in obesity-induced risks of cardiac remodeling. However, such suggestion has been based on a small number of 1–12 weeks of investigations that is insufficient for inducing a long-term adaptive physiological reaction in individual with obesity. Based on this, the term “HIIT” may not be appropriate in the absence of training “T" long-term adaptive response and could possibly be supplanted with “HI” in obesity (Alkhatib, 2023). One recent evidence indicated that 8 weeks of high-intensity interval cycling substantially elevated the glucose-insulin cycle and metabolic endotoxicity (1 session to 18 sessions equivalent to 24 sessions) in women (aged 18–30 years) with severe obesity (body fat>40%) (Alkhatib et al., 2020). Another observation also suggested that accumulated HIIT sessions might induce transient impairments in mitochondrial respiration and glucose homeostasis in healthy individuals (Flockhart et al., 2021). The negative acute metabolic response in the early to mid-phase elicited by HIIT might be ameliorated by prolonged adherence and adaption of high-intensity training. Current studies report outcomes after short-term of HIIT interventions, which highlighting the need for extended comprehensive follow-up, that is, months-to-years post rehabilitation. Actually, long-term adherence of HIIT is a challenging goal to reach in obesity with high-risk complications (Alkhatib, 2023). Additionally, high-intensity exercise with an associated high-intensity response temporarily increases the risk of precipitating a cardiac event in individuals with known or occult cardiovascular disease (Quindry et al., 2019; Alkhatib, 2023). According to a seminal study, high-intensity vigorous exercise increased the risk of acute cardiac events in patients with coronary heart disease by almost sixfold when compared to moderate-intensity exercise (Rognmo et al., 2012). Not surprisingly, no previously published studies have enrolled high-risk patients with obesity in HIIT intervention, and the dates of these publication indicate a migration from low-to moderate-risk participants. In particular, the majority of published studies enrolled low-risk individuals with obesity. Additional investigations should identify the specifics of the HIIT stimulus in patients with severe obesity-induced cardiac deterioration. Moreover, risk stratification of patients should be considered in relation to the appropriateness of HIIT rehabilitation. Another evidence from one research of home-based HIIT, in which one patient perished during the warm-up session, highlights the risk of acute cardiac events and the significance of direct patient supervision during exercise (Moholdt et al., 2012). While participation in cardiac rehabilitation under medical supervision significantly reduces the likelihood of subsequent cardiac events (Quindry et al., 2019), HIIT applications in medically supervised settings should also be addressed and investigated in obesity. Obviously, safety, long-term benefits, compliance, and harmful physiological side effects of HIIT implementation in individuals with obesity are still up for dispute. Thus, additional research is necessary to characterize the metabolic profiles and adaptive cardiometabolic responses of the HIIT stimulus in obesity for the accurately prescribe personalized lifestyle interventions (Alkhatib, 2023).

## 8 Conclusion

Given its robust effects on the adipose tissue distribution, inflammation, IR, and lipid metabolism, we would predict that HIIT would have a positive role against cardiac detrimental remodeling and disease caused by obesity or excess weight. Moreover, when compared with MICT, HIIT had a more significant effect on improving AT distribution (Honkala et al., 2017; Jo et al., 2020), fat oxidation (Vaccari et al., 2020), inflammatory reaction (Robinson et al., 2015; de Souza et al., 2018; Dorneles et al., 2019), and IR (Sun et al., 2019) in obesity-induced cardiac damage. Thus, HIIT can be viewed as a time-efficient intervention for managing individuals with overweight and obesity due to its equivalent efficacy and less time consumption to MICT. However, the physiological and molecular mechanisms of HIIT intervention in obesity, like energy metabolism, mitochondrial function, inflammatory reaction, insulin sensitivity, and other regulatory factors, require further study. Moreover, there was some indication that the adult weight status affected the efficacy of HIIT in reducing cardiometabolic disease risk (Batacan et al., 2017; Campbell et al., 2019). Adults classified as overweight or obese are more receptive to the benefits of HIIT than adults classified as average weight. In addition, compared to traditional MICT and resistance training, the regimes of HIIT are more complex and variable, such as having an increased intensity, work-to-rest ratio, or total duration. Thus, future studies should focus on investigating the differential response to HIIT treatment as a function of subject characteristics. As a result of the numerous advantages of HIIT on the primary elements of cardiometabolic health, a tailored exercise prescription should be more targeted in the treatment of individuals who are overweight or obese. Collective understanding highlights numerous specific research that are needed before the safety and effectiveness of HIIT can be confirmed and widely adopted in patient with obesity.

## References

[B1] Abassi W. Ouerghi N. Nikolaidis P. T. Hill L. Racil G. Knechtle B. (2021). Interval training with different intensities in overweight/obese adolescent females. Int. J. Sports Med. 43, 434–443. 10.1055/a-1648-4653 34749418

[B2] Addison O. Marcus R. L. Lastayo P. C. Ryan A. S. (2014). Intermuscular fat: A review of the consequences and causes. Int. J. Endocrinol. 2014, 309570. 10.1155/2014/309570 24527032 PMC3910392

[B3] Adir Y. Humbert M. Chaouat A. (2021). Sleep-related breathing disorders and pulmonary hypertension. Eur. Respir. J. 57 (1), 2002258. 10.1183/13993003.02258-2020 32747397

[B4] Aiad N. N. Hearon C. Jr. Hieda M. Dias K. Levine B. D. Sarma S. (2019). Mechanisms of left atrial enlargement in obesity. Am. J. Cardiol. 124 (3), 442–447. 10.1016/j.amjcard.2019.04.043 31133275

[B5] Alkhatib A. Hsieh M. J. Kuo C. H. Hou C. W. (2020). Caffeine optimizes HIIT benefits on obesity-associated metabolic adversity in women. Med. Sci. Sports Exerc 52 (8), 1793–1800. 10.1249/MSS.0000000000002311 32079912

[B6] Alkhatib A. (2023). Optimizing lifestyle behaviors in preventing multiple long-term conditions. Encyclopedia 3 (2), 468–477. 10.3390/encyclopedia3020032

[B7] Alvarez C. Ramirez-Campillo R. Ramirez-Velez R. Izquierdo M. (2017). Effects and prevalence of nonresponders after 12 weeks of high-intensity interval or resistance training in women with insulin resistance: A randomized trial. J. Appl. Physiol. (1985) 122 (4), 985–996. 10.1152/japplphysiol.01037.2016 28153946

[B8] Amano S. U. Cohen J. L. Vangala P. Tencerova M. Nicoloro S. M. Yawe J. C. (2014). Local proliferation of macrophages contributes to obesity-associated adipose tissue inflammation. Cell Metab. 19 (1), 162–171. 10.1016/j.cmet.2013.11.017 24374218 PMC3931314

[B9] Andreato L. V. (2020). High-intensity interval training: Methodological considerations for interpreting results and conducting research. Trends Endocrinol. Metab. 31 (11), 812–817. 10.1016/j.tem.2020.08.003 32967776

[B10] Arnett D. K. Blumenthal R. S. Albert M. A. Buroker A. B. Goldberger Z. D. Hahn E. J. (2019). 2019 ACC/AHA guideline on the primary prevention of cardiovascular disease: Executive summary: A report of the American College of cardiology/American heart association task force on clinical practice guidelines. Circulation 140 (11), e563–e595. 10.1161/CIR.0000000000000677 30879339 PMC8351755

[B11] Astani K. Bashiri J. Pourrazi H. Nourazar M. A. (2022). Effect of high-intensity interval training and coenzyme Q10 supplementation on cardiac apoptosis in obese male rats. ARYA Atheroscler. 18 (2), 1–9. 10.48305/arya.v18i0.2459 PMC9931613 36819840

[B12] Atakan M. M. Guzel Y. Shrestha N. Kosar S. N. Grgic J. Astorino T. A. (2022). Effects of high-intensity interval training (HIIT) and sprint interval training (SIT) on fat oxidation during exercise: A systematic review and meta-analysis. Br. J. Sports Med. 56, 988–996. 10.1136/bjsports-2021-105181 35859145

[B13] Avelar E. Cloward T. V. Walker J. M. Farney R. J. Strong M. Pendleton R. C. (2007). Left ventricular hypertrophy in severe obesity: Interactions among blood pressure, nocturnal hypoxemia, and body mass. Hypertension 49 (1), 34–39. 10.1161/01.HYP.0000251711.92482.14 17130310

[B14] Baek K. W. Lee D. I. Kang S. A. Yu H. S. (2020). Differences in macrophage polarization in the adipose tissue of obese mice under various levels of exercise intensity. J. Physiol. Biochem. 76 (1), 159–168. 10.1007/s13105-020-00731-7 32062818

[B15] Barry J. C. Simtchouk S. Durrer C. Jung M. E. Mui A. L. Little J. P. (2018). Short-term exercise training reduces anti-inflammatory action of interleukin-10 in adults with obesity. Cytokine 111, 460–469. 10.1016/j.cyto.2018.05.035 29885989

[B16] Batacan R. B. Jr. Duncan M. J. Dalbo V. J. Tucker P. S. Fenning A. S. (2017). Effects of high-intensity interval training on cardiometabolic health: A systematic review and meta-analysis of intervention studies. Br. J. Sports Med. 51 (6), 494–503. 10.1136/bjsports-2015-095841 27797726

[B17] Bellicha A. van Baak M. A. Battista F. Beaulieu K. Blundell J. E. Busetto L. (2021). Effect of exercise training on weight loss, body composition changes, and weight maintenance in adults with overweight or obesity: An overview of 12 systematic reviews and 149 studies. Obes. Rev. 22 (4), e13256. 10.1111/obr.13256 33955140 PMC8365736

[B18] Bermudez V. Duran P. Rojas E. Diaz M. P. Rivas J. Nava M. (2021). The sick adipose tissue: New insights into defective signaling and crosstalk with the myocardium. Front. Endocrinol. (Lausanne) 12, 735070. 10.3389/fendo.2021.735070 34603210 PMC8479191

[B19] Bluher S. Kapplinger J. Herget S. Reichardt S. Bottcher Y. Grimm A. (2017). Cardiometabolic risk markers, adipocyte fatty acid binding protein (aFABP) and the impact of high-intensity interval training (HIIT) in obese adolescents. Metabolism 68, 77–87. 10.1016/j.metabol.2016.11.015 28183455

[B20] Boardman N. T. Hafstad A. D. Lund J. Rossvoll L. Aasum E. (2017). Exercise of obese mice induces cardioprotection and oxygen sparing in hearts exposed to high-fat load. Am. J. Physiol. Heart Circ. Physiol. 313 (5), H1054–H1062. 10.1152/ajpheart.00382.2017 28801525

[B21] Boardman N. T. Rossvoll L. Lund J. Hafstad A. D. Aasum E. (2019). 3-Weeks of exercise training increases ischemic-tolerance in hearts from high-fat diet fed mice. Front. Physiol. 10, 1274. 10.3389/fphys.2019.01274 31632301 PMC6783811

[B22] Botta A. Liu Y. Wannaiampikul S. Tungtrongchitr R. Dadson K. Park T. S. (2019). An adiponectin-S1P axis protects against lipid induced insulin resistance and cardiomyocyte cell death via reduction of oxidative stress. Nutr. Metab. (Lond) 16, 14. 10.1186/s12986-019-0342-y 30828353 PMC6385438

[B23] Britton K. A. Massaro J. M. Murabito J. M. Kreger B. E. Hoffmann U. Fox C. S. (2013). Body fat distribution, incident cardiovascular disease, cancer, and all-cause mortality. J. Am. Coll. Cardiol. 62 (10), 921–925. 10.1016/j.jacc.2013.06.027 23850922 PMC4142485

[B24] Broussard J. L. Nelson M. D. Kolka C. M. Bediako I. A. Paszkiewicz R. L. Smith L. (2016). Rapid development of cardiac dysfunction in a canine model of insulin resistance and moderate obesity. Diabetologia 59 (1), 197–207. 10.1007/s00125-015-3767-5 26376797 PMC5310691

[B25] Buchheit M. Laursen P. B. (2013). High-intensity interval training, solutions to the programming puzzle: Part I: Cardiopulmonary emphasis. Sports Med. 43 (5), 313–338. 10.1007/s40279-013-0029-x 23539308

[B26] Bull F. C. Al-Ansari S. S. Biddle S. Borodulin K. Buman M. P. Cardon G. (2020). World Health Organization 2020 guidelines on physical activity and sedentary behaviour. Br. J. Sports Med. 54 (24), 1451–1462. 10.1136/bjsports-2020-102955 33239350 PMC7719906

[B27] Campbell W. W. Kraus W. E. Powell K. E. Haskell W. L. Janz K. F. Jakicic J. M. (2019). High-intensity interval training for cardiometabolic disease prevention. Med. Sci. Sports Exerc 51 (6), 1220–1226. 10.1249/mss.0000000000001934 31095079 PMC6777577

[B28] Cao M. Tang Y. Zou Y. (2022). Integrating high-intensity interval training into a school setting improve body composition, cardiorespiratory fitness and physical activity in children with obesity: A randomized controlled trial. J. Clin. Med. 11 (18), 5436. 10.3390/jcm11185436 36143083 PMC9506281

[B29] Caron A. Lee S. Elmquist J. K. Gautron L. (2018). Leptin and brain-adipose crosstalks. Nat. Rev. Neurosci. 19 (3), 153–165. 10.1038/nrn.2018.7 29449715 PMC5962962

[B30] Chahal H. McClelland R. L. Tandri H. Jain A. Turkbey E. B. Hundley W. G. (2012). Obesity and right ventricular structure and function: The MESA-right ventricle study. Chest 141 (2), 388–395. 10.1378/chest.11-0172 21868467 PMC3277293

[B31] Chien S. C. Chandramouli C. Lo C. I. Lin C. F. Sung K. T. Huang W. H. (2021). Associations of obesity and malnutrition with cardiac remodeling and cardiovascular outcomes in asian adults: A cohort study. PLoS Med. 18 (6), e1003661. 10.1371/journal.pmed.1003661 34061848 PMC8205172

[B32] Christensen R. H. Wedell-Neergaard A. S. Lehrskov L. L. Legaard G. E. Dorph E. Larsen M. K. (2019). Effect of aerobic and resistance exercise on cardiac adipose tissues: Secondary analyses from a randomized clinical trial. JAMA Cardiol. 4 (8), 778–787. 10.1001/jamacardio.2019.2074 31268469 PMC6613292

[B33] Collaborators G. B. D. O. Afshin A. Forouzanfar M. H. Reitsma M. B. Sur P. Estep K. (2017). Health effects of overweight and obesity in 195 countries over 25 years. N. Engl. J. Med. 377 (1), 13–27. 10.1056/NEJMoa1614362 28604169 PMC5477817

[B34] Czech M. P. (2017). Insulin action and resistance in obesity and type 2 diabetes. Nat. Med. 23 (7), 804–814. 10.1038/nm.4350 28697184 PMC6048953

[B35] de Castro-de-Paiva P. Marinho T. S. Mandarim-de-Lacerda C. A. Aguila M. B. (2022). Intermittent fasting, high-intensity interval training, or a combination of both have beneficial effects in obese mice with nonalcoholic fatty liver disease. J. Nutr. Biochem. 104, 108997. 10.1016/j.jnutbio.2022.108997 35331900

[B36] de Oliveira Sa G. Dos Santos Neves V. de Oliveira Fraga S. R. Souza-Mello V. Barbosa-da-Silva S. (2017). High-intensity interval training has beneficial effects on cardiac remodeling through local renin-angiotensin system modulation in mice fed high-fat or high-fructose diets. Life Sci. 189, 8–17. 10.1016/j.lfs.2017.09.012 28893641

[B37] de Souza D. C. Matos V. A. F. Dos Santos V. O. A. Medeiros I. F. Marinho C. S. R. Nascimento P. R. P. (2018). Effects of high-intensity interval and moderate-intensity continuous exercise on inflammatory, leptin, IgA, and lipid peroxidation responses in obese males. Front. Physiol. 9, 567. 10.3389/fphys.2018.00567 29875681 PMC5974092

[B39] Despres J. P. Carpentier A. C. Tchernof A. Neeland I. J. Poirier P. (2021). Management of obesity in cardiovascular practice: JACC focus seminar. J. Am. Coll. Cardiol. 78 (5), 513–531. 10.1016/j.jacc.2021.05.035 34325840 PMC8609918

[B40] Diniz T. A. Antunes B. M. Little J. P. Lira F. S. Rosa-Neto J. C. (2022). Exercise training protocols to improve obesity, glucose homeostasis, and subclinical inflammation. Methods Mol. Biol. 2343, 119–145. 10.1007/978-1-0716-1558-4_7 34473318

[B41] Dorneles G. P. da Silva I. Boeira M. C. Valentini D. Fonseca S. G. Dal Lago P. (2019). Cardiorespiratory fitness modulates the proportions of monocytes and T helper subsets in lean and obese men. Scand. J. Med. Sci. Sports 29 (11), 1755–1765. 10.1111/sms.13506 31241790

[B42] Dorneles G. P. Haddad D. O. Fagundes V. O. Vargas B. K. Kloecker A. Romao P. R. (2016). High intensity interval exercise decreases IL-8 and enhances the immunomodulatory cytokine interleukin-10 in lean and overweight-obese individuals. Cytokine 77, 1–9. 10.1016/j.cyto.2015.10.003 26476404

[B43] Dun Y. Smith J. R. Liu S. Olson T. P. (2019). High-intensity interval training in cardiac rehabilitation. Clin. Geriatr. Med. 35 (4), 469–487. 10.1016/j.cger.2019.07.011 31543179 PMC6760312

[B44] Eckel R. H. Jakicic J. M. Ard J. D. de Jesus J. M. Houston Miller N. Hubbard V. S. (2014). 2013 AHA/ACC guideline on lifestyle management to reduce cardiovascular risk: A report of the American College of cardiology/American heart association task force on practice guidelines. Circulation 129 (2), S76–S99. 10.1161/01.cir.0000437740.48606.d1 24222015

[B45] Eijsvogels T. M. Molossi S. Lee D. C. Emery M. S. Thompson P. D. (2016). Exercise at the extremes: The amount of exercise to reduce cardiovascular events. J. Am. Coll. Cardiol. 67 (3), 316–329. 10.1016/j.jacc.2015.11.034 26796398

[B46] Elsanhoury A. Nelki V. Kelle S. Van Linthout S. Tschope C. (2021). Epicardial fat expansion in diabetic and obese patients with heart failure and preserved ejection fraction-A specific HFpEF phenotype. Front. Cardiovasc Med. 8, 720690. 10.3389/fcvm.2021.720690 34604353 PMC8484763

[B47] Eskelinen J. J. Heinonen I. Loyttyniemi E. Hakala J. Heiskanen M. A. Motiani K. K. (2016). Left ventricular vascular and metabolic adaptations to high-intensity interval and moderate intensity continuous training: A randomized trial in healthy middle-aged men. J. Physiol. 594 (23), 7127–7140. 10.1113/JP273089 27500951 PMC5134384

[B48] Feng T. Vegard M. Strand L. B. Laugsand L. E. Morkedal B. Aune D. (2019). Weight and weight change and risk of atrial fibrillation: The HUNT study. Eur. Heart J. 40 (34), 2859–2866. 10.1093/eurheartj/ehz390 31209455

[B49] Finocchiaro G. Papadakis M. Dhutia H. Cole D. Behr E. R. Tome M. (2018). Obesity and sudden cardiac death in the young: Clinical and pathological insights from a large national registry. Eur. J. Prev. Cardiol. 25 (4), 395–401. 10.1177/2047487317751291 29319343

[B50] Fletcher G. F. Ades P. A. Kligfield P. Arena R. Balady G. J. Bittner V. A. (2013). Exercise standards for testing and training: A scientific statement from the American heart association. Circulation 128 (8), 873–934. 10.1161/CIR.0b013e31829b5b44 23877260

[B51] Flockhart M. Nilsson L. C. Tais S. Ekblom B. Apro W. Larsen F. J. (2021). Excessive exercise training causes mitochondrial functional impairment and decreases glucose tolerance in healthy volunteers. Cell Metab. 33 (5), 957–970.e6. 10.1016/j.cmet.2021.02.017 33740420

[B52] Fontanella R. A. Scisciola L. Rizzo M. R. Surina S. Sardu C. Marfella R. (2021). Adiponectin related vascular and cardiac benefits in obesity: Is there a role for an epigenetically regulated mechanism? Front. Cardiovasc Med. 8, 768026. 10.3389/fcvm.2021.768026 34869683 PMC8639875

[B53] Franca G. O. Frantz E. D. C. Magliano D. C. Bargut T. C. L. Sepulveda-Fragoso V. Silvares R. R. (2020). Effects of short-term high-intensity interval and continuous exercise training on body composition and cardiac function in obese sarcopenic rats. Life Sci. 256, 117920. 10.1016/j.lfs.2020.117920 32522571

[B54] Franklin B. A. Thompson P. D. Al-Zaiti S. S. Albert C. M. Hivert M. F. Levine B. D. (2020). Exercise-related acute cardiovascular events and potential deleterious adaptations following long-term exercise training: Placing the risks into perspective-an update: A scientific statement from the American heart association. Circulation 141 (13), e705–e736. 10.1161/CIR.0000000000000749 32100573

[B55] Fredrickson G. Barrow F. Dietsche K. Parthiban P. Khan S. Robert S. (2021). Exercise of high intensity ameliorates hepatic inflammation and the progression of NASH. Mol. Metab. 53, 101270. 10.1016/j.molmet.2021.101270 34118476 PMC8255932

[B56] Fumagalli C. Maurizi N. Day S. M. Ashley E. A. Michels M. Colan S. D. (2020). Association of obesity with adverse long-term outcomes in hypertrophic cardiomyopathy. JAMA Cardiol. 5 (1), 65–72. 10.1001/jamacardio.2019.4268 31693057 PMC6865784

[B57] Goodpaster B. H. Bergman B. C. Brennan A. M. Sparks L. M. (2023). Intermuscular adipose tissue in metabolic disease. Nat. Rev. Endocrinol. 19 (5), 285–298. 10.1038/s41574-022-00784-2 36564490

[B58] Gripp F. Nava R. C. Cassilhas R. C. Esteves E. A. Magalhaes C. O. D. Dias-Peixoto M. F. (2021). HIIT is superior than MICT on cardiometabolic health during training and detraining. Eur. J. Appl. Physiol. 121 (1), 159–172. 10.1007/s00421-020-04502-6 33000332

[B59] Guazzi M. Adams V. Conraads V. Halle M. Mezzani A. Vanhees L. (2012). EACPR/AHA Scientific Statement. Clinical recommendations for cardiopulmonary exercise testing data assessment in specific patient populations. Circulation 126 (18), 2261–2274. 10.1161/CIR.0b013e31826fb946 22952317 PMC4777325

[B60] Guzik T. J. Skiba D. S. Touyz R. M. Harrison D. G. (2017). The role of infiltrating immune cells in dysfunctional adipose tissue. Cardiovasc Res. 113 (9), 1009–1023. 10.1093/cvr/cvx108 28838042 PMC5852626

[B61] Hafstad A. D. Lund J. Hadler-Olsen E. Hoper A. C. Larsen T. S. Aasum E. (2013). High- and moderate-intensity training normalizes ventricular function and mechanoenergetics in mice with diet-induced obesity. Diabetes 62 (7), 2287–2294. 10.2337/db12-1580 23493573 PMC3712042

[B62] Hearon C. M. Jr. Dias K. A. MacNamara J. P. Hieda M. Mantha Y. Harada R. (2022). 1 Year HIIT and omega-3 fatty acids to improve cardiometabolic risk in stage-A heart failure. JACC Heart Fail 10 (4), 238–249. 10.1016/j.jchf.2022.01.004 35361442

[B63] Heiston E. M. Eichner N. Z. Gilbertson N. M. Malin S. K. (2020). Exercise improves adiposopathy, insulin sensitivity and metabolic syndrome severity independent of intensity. Exp. Physiol. 105 (4), 632–640. 10.1113/EP088158 32020676

[B64] Hingorani A. D. Finan C. Schmidt A. F. (2020). Obesity causes cardiovascular diseases: Adding to the weight of evidence. Eur. Heart J. 41 (2), 227–230. 10.1093/eurheartj/ehz569 31504435

[B65] Hirose K. Nakanishi K. Daimon M. Sawada N. Yoshida Y. Iwama K. (2021). Impact of insulin resistance on subclinical left ventricular dysfunction in normal weight and overweight/obese Japanese subjects in a general community. Cardiovasc Diabetol. 20 (1), 22. 10.1186/s12933-020-01201-6 33478525 PMC7818760

[B66] Honkala S. M. Motiani K. K. Eskelinen J. J. Savolainen A. Saunavaara V. Virtanen K. A. (2017). Exercise training reduces intrathoracic fat regardless of defective glucose tolerance. Med. Sci. Sports Exerc 49 (7), 1313–1322. 10.1249/MSS.0000000000001232 28628064 PMC5473372

[B67] Hulsmans M. Sager H. B. Roh J. D. Valero-Munoz M. Houstis N. E. Iwamoto Y. (2018). Cardiac macrophages promote diastolic dysfunction. J. Exp. Med. 215 (2), 423–440. 10.1084/jem.20171274 29339450 PMC5789416

[B68] Iacobellis G. (2015). Local and systemic effects of the multifaceted epicardial adipose tissue depot. Nat. Rev. Endocrinol. 11 (6), 363–371. 10.1038/nrendo.2015.58 25850659

[B69] Javaheri S. Barbe F. Campos-Rodriguez F. Dempsey J. A. Khayat R. Javaheri S. (2017). Sleep apnea: Types, mechanisms, and clinical cardiovascular consequences. J. Am. Coll. Cardiol. 69 (7), 841–858. 10.1016/j.jacc.2016.11.069 28209226 PMC5393905

[B70] Jensen M. D. Ryan D. H. Apovian C. M. Ard J. D. Comuzzie A. G. Donato K. A. (2014). 2013 AHA/ACC/TOS guideline for the management of overweight and obesity in adults: A report of the American College of cardiology/American heart association task force on practice guidelines and the obesity society. Circulation 129 (2), S102–S138. 10.1161/01.cir.0000437739.71477.ee 24222017 PMC5819889

[B71] Ji Y. Sun S. Shrestha N. Darragh L. B. Shirakawa J. Xing Y. (2019). Toll-like receptors TLR2 and TLR4 block the replication of pancreatic beta cells in diet-induced obesity. Nat. Immunol. 20 (6), 677–686. 10.1038/s41590-019-0396-z 31110312 PMC6531334

[B72] Jo E. A. Cho K. I. Park J. J. Im D. S. Choi J. H. Kim B. J. (2020). Effects of high-intensity interval training versus moderate-intensity continuous training on epicardial fat thickness and endothelial function in hypertensive metabolic syndrome. Metab. Syndr. Relat. Disord. 18 (2), 96–102. 10.1089/met.2018.0128 31928506

[B73] Joisten N. Gehlert S. Zimmer P. (2022). Is high-intensity interval training harmful to health? Trends Endocrinol. Metab. 33 (2), 85–86. 10.1016/j.tem.2021.07.003 34332852

[B74] Jordan A. S. McSharry D. G. Malhotra A. (2014). Adult obstructive sleep apnoea. Lancet 383 (9918), 736–747. 10.1016/S0140-6736(13)60734-5 23910433 PMC3909558

[B75] Kanazawa H. Yamabe H. Enomoto K. Koyama J. Morihisa K. Hoshiyama T. (2014). Importance of pericardial fat in the formation of complex fractionated atrial electrogram region in atrial fibrillation. Int. J. Cardiol. 174 (3), 557–564. 10.1016/j.ijcard.2014.04.135 24834998

[B76] Kao H. H. Hsu H. S. Wu T. H. Chiang H. F. Huang H. Y. Wang H. J. (2021). Effects of a single bout of short-duration high-intensity and long-duration low-intensity exercise on insulin resistance and adiponectin/leptin ratio. Obes. Res. Clin. Pract. 15 (1), 58–63. 10.1016/j.orcp.2020.09.007 33272841

[B77] Kenchaiah S. Ding J. Carr J. J. Allison M. A. Budoff M. J. Tracy R. P. (2021). Pericardial fat and the risk of heart failure. J. Am. Coll. Cardiol. 77 (21), 2638–2652. 10.1016/j.jacc.2021.04.003 34045020 PMC8218602

[B78] Kim J. S. Kim S. W. Lee J. S. Lee S. K. Abbott R. Lee K. Y. (2021). Association of pericardial adipose tissue with left ventricular structure and function: A region-specific effect? Cardiovasc Diabetol. 20 (1), 26. 10.1186/s12933-021-01219-4 33494780 PMC7836147

[B79] Kim K. Ahn N. Jung S. (2018). Comparison of endoplasmic reticulum stress and mitochondrial biogenesis responses after 12 weeks of treadmill running and ladder climbing exercises in the cardiac muscle of middle-aged obese rats. Braz J. Med. Biol. Res. 51 (10), e7508. 10.1590/1414-431X20187508 30066723 PMC6075797

[B80] Kim K. Ahn N. Jung S. Park S. (2017). Effects of intermittent ladder-climbing exercise training on mitochondrial biogenesis and endoplasmic reticulum stress of the cardiac muscle in obese middle-aged rats. Korean J. Physiol. Pharmacol. 21 (6), 633–641. 10.4196/kjpp.2017.21.6.633 29200906 PMC5709480

[B81] Kishi S. Gidding S. S. Reis J. P. Colangelo L. A. Venkatesh B. A. Armstrong A. C. (2017). Association of insulin resistance and glycemic metabolic abnormalities with LV structure and function in middle age: The CARDIA study. JACC Cardiovasc Imaging 10 (2), 105–114. 10.1016/j.jcmg.2016.02.033 27544896 PMC11961232

[B82] Kolahdouzi S. Talebi-Garakani E. Hamidian G. Safarzade A. (2019). Exercise training prevents high-fat diet-induced adipose tissue remodeling by promoting capillary density and macrophage polarization. Life Sci. 220, 32–43. 10.1016/j.lfs.2019.01.037 30690082

[B83] Koliaki C. Liatis S. Kokkinos A. (2019). Obesity and cardiovascular disease: Revisiting an old relationship. Metabolism 92, 98–107. 10.1016/j.metabol.2018.10.011 30399375

[B84] Kondamudi N. Thangada N. Patel K. V. Ayers C. Chandra A. Berry J. D. (2021). Regional adiposity, cardiorespiratory fitness, and left ventricular strain: An analysis from the dallas heart study. J. Cardiovasc Magn. Reson 23 (1), 78. 10.1186/s12968-021-00757-w 34120624 PMC8201708

[B85] Kondo H. Abe I. Gotoh K. Fukui A. Takanari H. Ishii Y. (2018). Interleukin 10 treatment ameliorates high-fat diet-induced inflammatory atrial remodeling and fibrillation. Circ. Arrhythm. Electrophysiol. 11 (5), e006040. 10.1161/CIRCEP.117.006040 29748196

[B86] Kong Z. Fan X. Sun S. Song L. Shi Q. Nie J. (2016a). Comparison of high-intensity interval training and moderate-to-vigorous continuous training for cardiometabolic health and exercise enjoyment in obese young women: A randomized controlled trial. PLoS One 11 (7), e0158589. 10.1371/journal.pone.0158589 27368057 PMC4930190

[B87] Kong Z. Shi Q. Nie J. Tong T. K. Song L. Yi L. (2017). High-intensity interval training in normobaric hypoxia improves cardiorespiratory fitness in overweight Chinese young women. Front. Physiol. 8, 175. 10.3389/fphys.2017.00175 28386234 PMC5362639

[B88] Kong Z. Sun S. Liu M. Shi Q. (2016b). Short-term high-intensity interval training on body composition and blood glucose in overweight and obese young women. J. Diabetes Res. 2016, 4073618. 10.1155/2016/4073618 27774458 PMC5059579

[B89] La Gerche A. Heidbuchel H. (2014). Can intensive exercise harm the heart? You can get too much of a good thing. Circulation 130 (12), 992–1002. 10.1161/circulationaha.114.008141 25223770

[B90] Larabee C. M. Neely O. C. Domingos A. I. (2020). Obesity: A neuroimmunometabolic perspective. Nat. Rev. Endocrinol. 16 (1), 30–43. 10.1038/s41574-019-0283-6 31776456

[B91] Lavie C. J. Arena R. Alpert M. A. Milani R. V. Ventura H. O. (2018). Management of cardiovascular diseases in patients with obesity. Nat. Rev. Cardiol. 15 (1), 45–56. 10.1038/nrcardio.2017.108 28748957

[B92] Le Jemtel T. H. Samson R. Ayinapudi K. Singh T. Oparil S. (2019). Epicardial adipose tissue and cardiovascular disease. Curr. Hypertens. Rep. 21 (5), 36. 10.1007/s11906-019-0939-6 30953236

[B93] Leake I. (2019). ANT2 mediates hypoxia and inflammation in obesity. Nat. Rev. Endocrinol. 15 (2), 64. 10.1038/s41574-018-0140-z 30531919

[B94] Lee C. H. Lui D. T. W. Lam K. S. L. (2021). Adipocyte fatty acid-binding protein, cardiovascular diseases and mortality. Front. Immunol. 12, 589206. 10.3389/fimmu.2021.589206 33815359 PMC8017191

[B95] Lee T. C. Jin Z. Homma S. Nakanishi K. Elkind M. S. V. Rundek T. (2019). Changes in left ventricular mass and geometry in the older adults: Role of body mass and central obesity. J. Am. Soc. Echocardiogr. 32 (10), 1318–1325. 10.1016/j.echo.2019.05.018 31311705 PMC6779504

[B96] Lee Y. S. Olefsky J. (2021). Chronic tissue inflammation and metabolic disease. Genes Dev. 35 (5-6), 307–328. 10.1101/gad.346312.120 33649162 PMC7919414

[B97] Levinger I. Shaw C. S. Stepto N. K. Cassar S. McAinch A. J. Cheetham C. (2015). What doesn't kill you makes you fitter: A systematic review of high-intensity interval exercise for patients with cardiovascular and metabolic diseases. Clin. Med. Insights Cardiol. 9, 53–63. 10.4137/CMC.S26230 PMC4482383 26157337

[B98] Lewis A. J. M. Abdesselam I. Rayner J. J. Byrne J. Borlaug B. A. Neubauer S. (2021). Adverse right ventricular remodelling, function, and stress responses in obesity: Insights from cardiovascular magnetic resonance. Eur. Heart J. Cardiovasc Imaging 23, 1383–1390. 10.1093/ehjci/jeab175 PMC9463995 34453521

[B99] Li X. Zhang D. Vatner D. F. Goedeke L. Hirabara S. M. Zhang Y. (2020). Mechanisms by which adiponectin reverses high fat diet-induced insulin resistance in mice. Proc. Natl. Acad. Sci. U. S. A. 117 (51), 32584–32593. 10.1073/pnas.1922169117 33293421 PMC7768680

[B100] Litwin S. E. Adams T. D. Davidson L. E. McKinlay R. Simper S. C. Ranson L. (2020). Longitudinal changes in cardiac structure and function in severe obesity: 11-Year follow-up in the Utah obesity study. J. Am. Heart Assoc. 9 (12), e014542. 10.1161/JAHA.119.014542 32476544 PMC7429060

[B101] Liu D. Huang Y. Huang C. Yang S. Wei X. Zhang P. (2022). Calorie restriction with or without time-restricted eating in weight loss. N. Engl. J. Med. 386 (16), 1495–1504. 10.1056/NEJMoa2114833 35443107

[B102] Lund J. Hafstad A. D. Boardman N. T. Rossvoll L. Rolim N. P. Ahmed M. S. (2015). Exercise training promotes cardioprotection through oxygen-sparing action in high fat-fed mice. Am. J. Physiol. Heart Circ. Physiol. 308 (8), H823–H829. 10.1152/ajpheart.00734.2014 25637547

[B103] Maeda N. Funahashi T. Matsuzawa Y. Shimomura I. (2020). Adiponectin, a unique adipocyte-derived factor beyond hormones. Atherosclerosis 292, 1–9. 10.1016/j.atherosclerosis.2019.10.021 31731079

[B104] Maffetone P. B. Rivera-Dominguez I. Laursen P. B. (2016). Overfat and underfat: New terms and definitions long overdue. Front. Public Health 4, 279. 10.3389/fpubh.2016.00279 28097119 PMC5206235

[B105] Mandry D. Girerd N. Lamiral Z. Huttin O. Filippetti L. Micard E. (2021). Arterial and cardiac remodeling associated with extra weight gain in an isolated abdominal obesity cohort. Front. Cardiovasc Med. 8, 771022. 10.3389/fcvm.2021.771022 34805324 PMC8602697

[B106] Martinez-Huenchullan S. F. Fox S. L. Tam C. S. Maharjan B. R. Olaya-Agudo L. F. Ehrenfeld P. (2020). Constant-moderate versus high-intensity interval training on heart adiponectin levels in high-fat fed mice: A preventive and treatment approach. Arch. Physiol. Biochem. 129, 41–45. 10.1080/13813455.2020.1797098 32715774

[B107] May H. T. Nelson J. R. Lirette S. T. Kulkarni K. R. Anderson J. L. Griswold M. E. (2016). The utility of the apolipoprotein A1 remnant ratio in predicting incidence coronary heart disease in a primary prevention cohort: The Jackson Heart Study. Eur. J. Prev. Cardiol. 23 (7), 769–776. 10.1177/2047487315612733 26481445 PMC5913735

[B108] McGregor G. Powell R. Begg B. Birkett S. T. Nichols S. Ennis S. (2023). High-intensity interval training in cardiac rehabilitation: A multi-centre randomized controlled trial. Eur. J. Prev. Cardiol. 30 (9), 745–755. 10.1093/eurjpc/zwad039 36753063

[B109] McManus D. D. Xanthakis V. Sullivan L. M. Zachariah J. Aragam J. Larson M. G. (2010). Longitudinal tracking of left atrial diameter over the adult life course: Clinical correlates in the community. Circulation 121 (5), 667–674. 10.1161/CIRCULATIONAHA.109.885806 20100973 PMC2823068

[B110] Mendelson M. Chacaroun S. Baillieul S. Doutreleau S. Guinot M. Wuyam B. (2022). Effects of high intensity interval training on sustained reduction in cardiometabolic risk associated with overweight/obesity. A randomized trial. J. Exerc Sci. Fit. 20 (2), 172–181. 10.1016/j.jesf.2022.03.001 35401768 PMC8956941

[B111] Moholdt T. Bekken Vold M. Grimsmo J. Slordahl S. A. Wisloff U. (2012). Home-based aerobic interval training improves peak oxygen uptake equal to residential cardiac rehabilitation: A randomized, controlled trial. PLoS One 7 (7), e41199. 10.1371/journal.pone.0041199 22815970 PMC3399826

[B112] Mora-Rodriguez R. Ramirez-Jimenez M. Fernandez-Elias V. E. Guio de Prada M. V. Morales-Palomo F. Pallares J. G. (2018). Effects of aerobic interval training on arterial stiffness and microvascular function in patients with metabolic syndrome. J. Clin. Hypertens. (Greenwich) 20 (1), 11–18. 10.1111/jch.13130 29106772 PMC8031296

[B113] Mouton A. J. Li X. Hall M. E. Hall J. E. (2020). Obesity, hypertension, and cardiac dysfunction: Novel roles of immunometabolism in macrophage activation and inflammation. Circ. Res. 126 (6), 789–806. 10.1161/CIRCRESAHA.119.312321 32163341 PMC7255054

[B114] Mozaffarian D. Furberg C. D. Psaty B. M. Siscovick D. (2008). Physical activity and incidence of atrial fibrillation in older adults: The cardiovascular health study. Circulation 118 (8), 800–807. 10.1161/CIRCULATIONAHA.108.785626 18678768 PMC3133958

[B115] Nakamura M. Sadoshima J. (2020). Cardiomyopathy in obesity, insulin resistance and diabetes. J. Physiol. 598 (14), 2977–2993. 10.1113/JP276747 30869158

[B116] Nalliah C. J. Bell J. R. Raaijmakers A. J. A. Waddell H. M. Wells S. P. Bernasochi G. B. (2020). Epicardial adipose tissue accumulation confers atrial conduction abnormality. J. Am. Coll. Cardiol. 76 (10), 1197–1211. 10.1016/j.jacc.2020.07.017 32883413

[B117] Neeland I. J. Ayers C. R. Rohatgi A. K. Turer A. T. Berry J. D. Das S. R. (2013). Associations of visceral and abdominal subcutaneous adipose tissue with markers of cardiac and metabolic risk in obese adults. Obes. (Silver Spring) 21 (9), E439–E447. 10.1002/oby.20135 PMC3751977 23687099

[B118] Neeland I. J. Poirier P. Despres J. P. (2018). Cardiovascular and metabolic heterogeneity of obesity: Clinical challenges and implications for management. Circulation 137 (13), 1391–1406. 10.1161/CIRCULATIONAHA.117.029617 29581366 PMC5875734

[B119] Neeland I. J. Ross R. Despres J. P. Matsuzawa Y. Yamashita S. Shai I. (2019). Visceral and ectopic fat, atherosclerosis, and cardiometabolic disease: A position statement. Lancet Diabetes Endocrinol. 7 (9), 715–725. 10.1016/S2213-8587(19)30084-1 31301983

[B120] Nes B. M. Janszky I. Wisløff U. Støylen A. Karlsen T. (2013). Age-predicted maximal heart rate in healthy subjects: The HUNT fitness study. Scand. J. Med. Sci. Sports 23 (6), 697–704. 10.1111/j.1600-0838.2012.01445.x 22376273

[B121] Nyawo T. A. Pheiffer C. Mazibuko-Mbeje S. E. Mthembu S. X. H. Nyambuya T. M. Nkambule B. B. (2021). Physical exercise potentially targets epicardial adipose tissue to reduce cardiovascular disease risk in patients with metabolic diseases: Oxidative stress and inflammation emerge as major therapeutic targets. Antioxidants (Basel) 10 (11), 1758. 10.3390/antiox10111758 34829629 PMC8614861

[B122] Paahoo A. Tadibi V. Behpoor N. (2021). Effectiveness of continuous aerobic versus high-intensity interval training on atherosclerotic and inflammatory markers in boys with overweight/obesity. Pediatr. Exerc Sci. 33 (3), 132–138. 10.1123/pes.2020-0138 33761458

[B123] Packer M. (2018). Epicardial adipose tissue may mediate deleterious effects of obesity and inflammation on the myocardium. J. Am. Coll. Cardiol. 71 (20), 2360–2372. 10.1016/j.jacc.2018.03.509 29773163

[B124] Park J. B. Kim D. H. Lee H. Hwang I. C. Yoon Y. E. Park H. E. (2020). Obesity and metabolic health status are determinants for the clinical expression of hypertrophic cardiomyopathy. Eur. J. Prev. Cardiol. 27 (17), 1849–1857. 10.1177/2047487319889714 31787021

[B125] Pedersen L. R. Olsen R. H. Anholm C. Astrup A. Eugen-Olsen J. Fenger M. (2019). Effects of 1 year of exercise training versus combined exercise training and weight loss on body composition, low-grade inflammation and lipids in overweight patients with coronary artery disease: A randomized trial. Cardiovasc Diabetol. 18 (1), 127. 10.1186/s12933-019-0934-x 31575375 PMC6774219

[B126] Peppard P. E. Young T. Palta M. Dempsey J. Skatrud J. (2000). Longitudinal study of moderate weight change and sleep-disordered breathing. JAMA 284 (23), 3015–3021. 10.1001/jama.284.23.3015 11122588

[B127] Petersen M. H. de Almeida M. E. Wentorf E. K. Jensen K. Ortenblad N. Hojlund K. (2022). High-intensity interval training combining rowing and cycling efficiently improves insulin sensitivity, body composition and VO(2)max in men with obesity and type 2 diabetes. Front. Endocrinol. (Lausanne) 13, 1032235. 10.3389/fendo.2022.1032235 36387850 PMC9664080

[B128] Phillips B. E. Kelly B. M. Lilja M. Ponce-Gonzalez J. G. Brogan R. J. Morris D. L. (2017). A practical and time-efficient high-intensity interval training program modifies cardio-metabolic risk factors in adults with risk factors for type II diabetes. Front. Endocrinol. (Lausanne) 8, 229. 10.3389/fendo.2017.00229 28943861 PMC5596071

[B129] Piche M. E. Tchernof A. Despres J. P. (2020). Obesity phenotypes, diabetes, and cardiovascular diseases. Circ. Res. 126 (11), 1477–1500. 10.1161/CIRCRESAHA.120.316101 32437302

[B130] Plavsic L. Knezevic O. M. Sovtic A. Minic P. Vukovic R. Mazibrada I. (2020). Effects of high-intensity interval training and nutrition advice on cardiometabolic markers and aerobic fitness in adolescent girls with obesity. Appl. Physiol. Nutr. Metab. 45 (3), 294–300. 10.1139/apnm-2019-0137 31386826

[B131] Poirier P. Giles T. D. Bray G. A. Hong Y. Stern J. S. Pi-Sunyer F. X. (2006). Obesity and cardiovascular disease: Pathophysiology, evaluation, and effect of weight loss. Arterioscler. Thromb. Vasc. Biol. 26 (5), 968–976. 10.1161/01.ATV.0000216787.85457.f3 16627822

[B132] Poon E. T. Siu P. M. Wongpipit W. Gibala M. Wong S. H. (2022). Alternating high-intensity interval training and continuous training is efficacious in improving cardiometabolic health in obese middle-aged men. J. Exerc Sci. Fit. 20 (1), 40–47. 10.1016/j.jesf.2021.11.003 34987589 PMC8689221

[B133] Powell-Wiley T. M. Poirier P. Burke L. E. Despres J. P. Gordon-Larsen P. Lavie C. J. (2021). Obesity and cardiovascular disease: A scientific statement from the American heart association. Circulation 143 (21), e984–e1010. 10.1161/CIR.0000000000000973 33882682 PMC8493650

[B134] Quindry J. C. Franklin B. A. Chapman M. Humphrey R. Mathis S. (2019). Benefits and risks of high-intensity interval training in patients with coronary artery disease. Am. J. Cardiol. 123 (8), 1370–1377. 10.1016/j.amjcard.2019.01.008 30732854

[B135] Racil G. Ben Ounis O. Hammouda O. Kallel A. Zouhal H. Chamari K. (2013). Effects of high vs. moderate exercise intensity during interval training on lipids and adiponectin levels in obese young females. Eur. J. Appl. Physiol. 113 (10), 2531–2540. 10.1007/s00421-013-2689-5 23824463

[B136] Racil G. Coquart J. B. Elmontassar W. Haddad M. Goebel R. Chaouachi A. (2016a). Greater effects of high-compared with moderate-intensity interval training on cardio-metabolic variables, blood leptin concentration and ratings of perceived exertion in obese adolescent females. Biol. Sport 33 (2), 145–152. 10.5604/20831862.1198633 27274107 PMC4885625

[B137] Racil G. Zouhal H. Elmontassar W. Ben Abderrahmane A. De Sousa M. V. Chamari K. (2016b). Plyometric exercise combined with high-intensity interval training improves metabolic abnormalities in young obese females more so than interval training alone. Appl. Physiol. Nutr. Metab. 41 (1), 103–109. 10.1139/apnm-2015-0384 26701117

[B138] Rafeh R. Viveiros A. Oudit G. Y. El-Yazbi A. F. (2020). Targeting perivascular and epicardial adipose tissue inflammation: Therapeutic opportunities for cardiovascular disease. Clin. Sci. (Lond) 134 (7), 827–851. 10.1042/CS20190227 32271386

[B139] Rahmati-Ahmadabad S. Azarbayjani M. A. Farzanegi P. Moradi L. (2019b). High-intensity interval training has a greater effect on reverse cholesterol transport elements compared with moderate-intensity continuous training in obese male rats. Eur. J. Prev. Cardiol. 28 (7), 692–701. 10.1177/2047487319887828 33611472

[B140] Rahmati-Ahmadabad S. Broom D. R. Ghanbari-Niaki A. Shirvani H. (2019a). Effects of exercise on reverse cholesterol transport: A systemized narrative review of animal studies. Life Sci. 224, 139–148. 10.1016/j.lfs.2019.03.058 30922848

[B141] Rajapurohitam V. Gan X. T. Kirshenbaum L. A. Karmazyn M. (2003). The obesity-associated peptide leptin induces hypertrophy in neonatal rat ventricular myocytes. Circ. Res. 93 (4), 277–279. 10.1161/01.Res.0000089255.37804.72 12893740

[B142] Ramos J. S. Dalleck L. C. Borrani F. Beetham K. S. Wallen M. P. Mallard A. R. (2017). Low-volume high-intensity interval training is sufficient to ameliorate the severity of metabolic syndrome. Metab. Syndr. Relat. Disord. 15 (7), 319–328. 10.1089/met.2017.0042 28846513

[B143] Ramos J. S. Dalleck L. C. Fennell M. Martini A. Welmans T. Stennett R. (2021). Exercise training intensity and the fitness-fatness index in adults with metabolic syndrome: A randomized trial. Sports Med. Open 7 (1), 100. 10.1186/s40798-021-00395-7 34951682 PMC8709799

[B144] Rao V. N. Fudim M. Mentz R. J. Michos E. D. Felker G. M. (2020). Regional adiposity and heart failure with preserved ejection fraction. Eur. J. Heart Fail 22 (9), 1540–1550. 10.1002/ejhf.1956 32619081 PMC9991865

[B145] Ratajczak M. Skrypnik D. Krutki P. Karolkiewicz J. (2020). Effects of an indoor cycling program on cardiometabolic factors in women with obesity vs. Normal body weight. Int. J. Environ. Res. Public Health 17 (23), 8718. 10.3390/ijerph17238718 33255278 PMC7727675

[B146] Reljic D. Dieterich W. Herrmann H. J. Neurath M. F. Zopf Y. (2022). HIIT the inflammation": Comparative effects of low-volume interval training and resistance exercises on inflammatory indices in obese metabolic syndrome patients undergoing caloric restriction. Nutrients 14 (10), 1996. 10.3390/nu14101996 35631137 PMC9145085

[B147] Reljic D. Frenk F. Herrmann H. J. Neurath M. F. Zopf Y. (2021b). Effects of very low volume high intensity versus moderate intensity interval training in obese metabolic syndrome patients: A randomized controlled study. Sci. Rep. 11 (1), 2836. 10.1038/s41598-021-82372-4 33531522 PMC7854610

[B148] Reljic D. Konturek P. C. Herrmann H. J. Siebler J. Neurath M. F. Zopf Y. (2021a). Very low-volume interval training improves nonalcoholic fatty liver disease fibrosis score and cardiometabolic health in adults with obesity and metabolic syndrome. J. Physiol. Pharmacol. 72 (6). 10.26402/jpp.2021.6.10 35485357

[B149] Reljic D. Lampe D. Wolf F. Zopf Y. Herrmann H. J. Fischer J. (2019). Prevalence and predictors of dropout from high-intensity interval training in sedentary individuals: A meta-analysis. Scand. J. Med. Sci. Sports 29 (9), 1288–1304. 10.1111/sms.13452 31050061

[B150] Ren J. Wu N. N. Wang S. Sowers J. R. Zhang Y. (2021). Obesity cardiomyopathy: Evidence, mechanisms, and therapeutic implications. Physiol. Rev. 101 (4), 1745–1807. 10.1152/physrev.00030.2020 33949876 PMC8422427

[B151] Riebe D. Ehrman J. K. Liguori G. Magal M. (Editors) (2018). ACSM’s guidelines for exercise testing and prescription. Tenth ed (Wolters Kluwer).

[B152] Riebe D. Franklin B. A. Thompson P. D. Garber C. E. Whitfield G. P. Magal M. (2015). Updating ACSM's recommendations for exercise preparticipation health screening. Med. Sci. Sports Exerc 47 (11), 2473–2479. 10.1249/mss.0000000000000664 26473759

[B153] Robertson J. Lindgren M. Schaufelberger M. Adiels M. Bjorck L. Lundberg C. E. (2020). Body mass index in young women and risk of cardiomyopathy: A long-term follow-up study in Sweden. Circulation 141 (7), 520–529. 10.1161/CIRCULATIONAHA.119.044056 32065765 PMC7017947

[B154] Robinson E. Durrer C. Simtchouk S. Jung M. E. Bourne J. E. Voth E. (2015). Short-term high-intensity interval and moderate-intensity continuous training reduce leukocyte TLR4 in inactive adults at elevated risk of type 2 diabetes. J. Appl. Physiol. (1985) 119 (5), 508–516. 10.1152/japplphysiol.00334.2015 26139217 PMC4556835

[B155] Rognmo O. Moholdt T. Bakken H. Hole T. Molstad P. Myhr N. E. (2012). Cardiovascular risk of high-versus moderate-intensity aerobic exercise in coronary heart disease patients. Circulation 126 (12), 1436–1440. 10.1161/CIRCULATIONAHA.112.123117 22879367

[B156] Sacks F. M. Liang L. Furtado J. D. Cai T. Davidson W. S. He Z. (2020). Protein-defined subspecies of HDLs (High-Density lipoproteins) and differential risk of coronary heart disease in 4 prospective studies. Arterioscler. Thromb. Vasc. Biol. 40 (11), 2714–2727. 10.1161/atvbaha.120.314609 32907368 PMC7577984

[B157] Saco-Ledo G. Valenzuela P. L. Castillo-Garcia A. Arenas J. Leon-Sanz M. Ruilope L. M. (2021). Physical exercise and epicardial adipose tissue: A systematic review and meta-analysis of randomized controlled trials. Obes. Rev. 22 (1), e13103. 10.1111/obr.13103 32692478

[B158] Savji N. Meijers W. C. Bartz T. M. Bhambhani V. Cushman M. Nayor M. (2018). The association of obesity and cardiometabolic traits with incident HFpEF and HFrEF. JACC Heart Fail 6 (8), 701–709. 10.1016/j.jchf.2018.05.018 30007554 PMC6076337

[B159] Sawada N. Nakanishi K. Daimon M. Yoshida Y. Ishiwata J. Hirokawa M. (2020). Influence of visceral adiposity accumulation on adverse left and right ventricular mechanics in the community. Eur. J. Prev. Cardiol. 27 (18), 2006–2015. 10.1177/2047487319891286 31795766

[B160] Seo J. B. Riopel M. Cabrales P. Huh J. Y. Bandyopadhyay G. K. Andreyev A. Y. (2019). Knockdown of Ant2 reduces adipocyte hypoxia and improves insulin resistance in obesity. Nat. Metab. 1 (1), 86–97. 10.1038/s42255-018-0003-x 31528845 PMC6746433

[B161] Smith G. I. Mittendorfer B. Klein S. (2019). Metabolically healthy obesity: Facts and fantasies. J. Clin. Invest. 129 (10), 3978–3989. 10.1172/JCI129186 31524630 PMC6763224

[B162] Sorimachi H. Obokata M. Takahashi N. Reddy Y. N. V. Jain C. C. Verbrugge F. H. (2021). Pathophysiologic importance of visceral adipose tissue in women with heart failure and preserved ejection fraction. Eur. Heart J. 42 (16), 1595–1605. 10.1093/eurheartj/ehaa823 33227126 PMC8060057

[B163] Stone N. J. Robinson J. G. Lichtenstein A. H. Bairey Merz C. N. Blum C. B. Eckel R. H. (2014). 2013 ACC/AHA guideline on the treatment of blood cholesterol to reduce atherosclerotic cardiovascular risk in adults: A report of the American College of cardiology/American heart association task force on practice guidelines. Circulation 129 (2), S1–S45. 10.1161/01.cir.0000437738.63853.7a 24222016

[B164] Straub L. G. Scherer P. E. (2019). Metabolic messengers: Adiponectin. Nat. Metab. 1 (3), 334–339. 10.1038/s42255-019-0041-z 32661510 PMC7357716

[B165] Sultana R. N. Sabag A. Keating S. E. Johnson N. A. (2019). The effect of low-volume high-intensity interval training on body composition and cardiorespiratory fitness: A systematic review and meta-analysis. Sports Med. 49 (11), 1687–1721. 10.1007/s40279-019-01167-w 31401727

[B166] Sun L. Li F. H. Li T. Min Z. Yang L. D. Gao H. E. (2020). Effects of high-intensity interval training on adipose tissue lipolysis, inflammation, and metabolomics in aged rats. Pflugers Arch. 472 (2), 245–258. 10.1007/s00424-020-02351-y 32006095

[B167] Sun S. Zhang H. Kong Z. Shi Q. Tong T. K. Nie J. (2019). Twelve weeks of low volume sprint interval training improves cardio-metabolic health outcomes in overweight females. J. Sports Sci. 37 (11), 1257–1264. 10.1080/02640414.2018.1554615 30563431

[B168] Taylor J. L. Bonikowske A. R. Olson T. P. (2021). Optimizing outcomes in cardiac rehabilitation: The importance of exercise intensity. Front. Cardiovasc Med. 8, 734278. 10.3389/fcvm.2021.734278 34540924 PMC8446279

[B169] Taylor J. L. Holland D. J. Spathis J. G. Beetham K. S. Wisløff U. Keating S. E. (2019). Guidelines for the delivery and monitoring of high intensity interval training in clinical populations. Prog. Cardiovasc Dis. 62 (2), 140–146. 10.1016/j.pcad.2019.01.004 30685470

[B170] Tong T. K. Zhang H. Shi H. Liu Y. Ai J. Nie J. (2018). Comparing time efficiency of sprint vs. High-intensity interval training in reducing abdominal visceral fat in obese young women: A randomized, controlled trial. Front. Physiol. 9, 1048. 10.3389/fphys.2018.01048 30123136 PMC6085472

[B171] Trayhurn P. (2013). Hypoxia and adipose tissue function and dysfunction in obesity. Physiol. Rev. 93 (1), 1–21. 10.1152/physrev.00017.2012 23303904

[B172] Tremblay A. Simoneau J. A. Bouchard C. (1994). Impact of exercise intensity on body fatness and skeletal muscle metabolism. Metabolism 43 (7), 814–818. 10.1016/0026-0495(94)90259-3 8028502

[B173] Vaccari F. Passaro A. D'Amuri A. Sanz J. M. Di Vece F. Capatti E. (2020). Effects of 3-month high-intensity interval training vs. moderate endurance training and 4-month follow-up on fat metabolism, cardiorespiratory function and mitochondrial respiration in obese adults. Eur. J. Appl. Physiol. 120 (8), 1787–1803. 10.1007/s00421-020-04409-2 32514607

[B174] Vardar S. A. Karaca A. Guldiken S. Palabiyik O. Sut N. Demir A. M. (2018). High-intensity interval training acutely alters plasma adipokine levels in young overweight/obese women. Arch. Physiol. Biochem. 124 (2), 149–155. 10.1080/13813455.2017.1369998 28857629

[B175] Vella C. A. Taylor K. Drummer D. (2017). High-intensity interval and moderate-intensity continuous training elicit similar enjoyment and adherence levels in overweight and obese adults. Eur. J. Sport Sci. 17 (9), 1203–1211. 10.1080/17461391.2017.1359679 28792851 PMC6104631

[B176] Viana R. B. Naves J. P. A. Coswig V. S. de Lira C. A. B. Steele J. Fisher J. P. (2019). Is interval training the magic bullet for fat loss? A systematic review and meta-analysis comparing moderate-intensity continuous training with high-intensity interval training (HIIT). Br. J. Sports Med. 53 (10), 655–664. 10.1136/bjsports-2018-099928 30765340

[B177] Vishvanath L. Gupta R. K. (2019). Contribution of adipogenesis to healthy adipose tissue expansion in obesity. J. Clin. Invest. 129 (10), 4022–4031. 10.1172/JCI129191 31573549 PMC6763245

[B178] von Haehling S. Doehner W. Anker S. D. (2019). The evolving obesity paradigm story: From heart failure to atrial fibrillation. Eur. Heart J. 40 (19), 1550–1552. 10.1093/eurheartj/ehz082 31009055

[B179] von Klot S. Mittleman M. A. Dockery D. W. Heier M. Meisinger C. Hormann A. (2008). Intensity of physical exertion and triggering of myocardial infarction: A case-crossover study. Eur. Heart J. 29 (15), 1881–1888. 10.1093/eurheartj/ehn235 18534976

[B180] Walczak-Galezewska M. K. Markowska M. Braszak A. Bryl W. Bogdanski P. (2019). Atrial fibrillation and obesity: Should doctors focus on this comorbidity? Minerva Med. 110 (2), 175–176. 10.23736/S0026-4806.18.05816-0 30334438

[B181] Weston K. S. Wisloff U. Coombes J. S. (2014). High-intensity interval training in patients with lifestyle-induced cardiometabolic disease: A systematic review and meta-analysis. Br. J. Sports Med. 48 (16), 1227–1234. 10.1136/bjsports-2013-092576 24144531

[B182] Wong C. X. Sullivan T. Sun M. T. Mahajan R. Pathak R. K. Middeldorp M. (2015). Obesity and the risk of incident, post-operative, and post-ablation atrial fibrillation: A meta-analysis of 626,603 individuals in 51 studies. JACC Clin. Electrophysiol. 1 (3), 139–152. 10.1016/j.jacep.2015.04.004 29759357

[B183] Wu H. Ballantyne C. M. (2020). Metabolic inflammation and insulin resistance in obesity. Circ. Res. 126 (11), 1549–1564. 10.1161/CIRCRESAHA.119.315896 32437299 PMC7250139

[B184] Xia X. Jung D. Webb P. Zhang A. Zhang B. Li L. (2012). Liver X receptor beta and peroxisome proliferator-activated receptor delta regulate cholesterol transport in murine cholangiocytes. Hepatology 56 (6), 2288–2296. 10.1002/hep.25919 22729460 PMC3469731

[B185] Xu E. Kachenoura N. Della Valle V. Dubern B. Karsenty A. Tounian P. (2021). Multichamber dysfunction in children and adolescents with severe obesity: A cardiac magnetic resonance imaging myocardial strain study. J. Magn. Reson Imaging 54 (5), 1393–1403. 10.1002/jmri.27796 34155711

[B186] Yang M. Qiu S. He Y. Li L. Wu T. Ding N. (2021). Genetic ablation of C-reactive protein gene confers resistance to obesity and insulin resistance in rats. Diabetologia 64 (5), 1169–1183. 10.1007/s00125-021-05384-9 33544171

[B187] Yeghiazarians Y. Jneid H. Tietjens J. R. Redline S. Brown D. L. El-Sherif N. (2021). Obstructive sleep apnea and cardiovascular disease: A scientific statement from the American heart association. Circulation 144 (3), e56–e67. 10.1161/CIR.0000000000000988 34148375

[B188] Yim J. E. Heshka S. Albu J. Heymsfield S. Kuznia P. Harris T. (2007). Intermuscular adipose tissue rivals visceral adipose tissue in independent associations with cardiovascular risk. Int. J. Obes. (Lond) 31 (9), 1400–1405. 10.1038/sj.ijo.0803621 17452994 PMC2752367

[B189] Youssef L. Granet J. Marcangeli V. Dulac M. Hajj-Boutros G. Reynaud O. (2022). Clinical and biological adaptations in obese older adults following 12-weeks of high-intensity interval training or moderate-intensity continuous training. Healthc. (Basel) 10 (7), 1346. 10.3390/healthcare10071346 PMC9315493 35885872

[B190] Zhang H. Tong T. K. Qiu W. Zhang X. Zhou S. Liu Y. (2017). Comparable effects of high-intensity interval training and prolonged continuous exercise training on abdominal visceral fat reduction in obese young women. J. Diabetes Res. 2017, 5071740. 10.1155/2017/5071740 28116314 PMC5237463

[B191] Zhao S. Kusminski C. M. Scherer P. E. (2021). Adiponectin, leptin and cardiovascular disorders. Circ. Res. 128 (1), 136–149. 10.1161/CIRCRESAHA.120.314458 33411633 PMC7799441

[B192] Zhu L. Liu J. Yu Y. Tian Z. (2021). Effect of high-intensity interval training on cardiometabolic risk factors in childhood obesity: A meta-analysis. J. Sports Med. Phys. Fit. 61 (5), 743–752. 10.23736/S0022-4707.20.11329-X 33975429

